# GFI1B acts as a metabolic regulator in hematopoiesis and acute myeloid leukemia

**DOI:** 10.1038/s41375-022-01635-9

**Published:** 2022-07-08

**Authors:** Longlong Liu, Pradeep Kumar Patnana, Xiaoqing Xie, Daria Frank, Subbaiah Chary Nimmagadda, Minhua Su, Donghua Zhang, Thorsten Koenig, Frank Rosenbauer, Marie Liebmann, Luisa Klotz, Wendan Xu, Jan Vorwerk, Felix Neumann, Jana Hüve, Andreas Unger, Jürgen Günther Okun, Bertram Opalka, Cyrus Khandanpour

**Affiliations:** 1 Department of Medicine A, Hematology, Oncology and Pneumology, University Hospital Muenster, 48149 Muenster, Germany; 2 Department of Hematology and Stem Cell Transplantation, University Hospital Essen, University of Duisburg-Essen, 45147 Essen, Germany; 3 State Key Laboratory of Experimental Hematology, National Clinical Research Center for Blood Diseases, Haihe Laboratory of Cell Ecosystem, Institute of Hematology & Blood Diseases Hospital, Chinese Academy of Medical Sciences & Peking Union Medical College, 300052 Tianjin, China; 4 Department of Hematology, Tongji Hospital of Tongji Medical College of Huazhong University of Science and Technology, 430030 Wuhan, China; 5 Institute of Molecular Tumor Biology, Faculty of Medicine, University of Muenster, 48149 Muenster, Germany; 6 Department of Neurology with Institute of Translational Neurology, University Hospital Muenster, 48149 Muenster, Germany; 7 Fluorescence Microscopy Facility Muenster (FM)2, Institute of Medical Physics and Biophysics, University of Muenster, 48149 Muenster, Germany; 8 evorion biotechnologies GmbH, 48149 Muenster, Germany; 9 Institute of Physiology II, University of Muenster, 48149 Muenster, Germany; 10 Department of General Pediatrics, Division of Neuropediatrics and Metabolic Medicine, Dietmar-Hopp-Metabolic Center, 69120 Heidelberg, Germany; 11 Department of Hematology and Oncology, University Hospital of Schleswig-Holstein, University of Luebeck, 23538 Luebeck, Germany

**Keywords:** Cancer metabolism, Acute myeloid leukaemia, Haematopoiesis, Drug development

## Abstract

Recent studies highlighted the role of transcription factors in metabolic regulation during hematopoiesis and leukemia development. GFI1B is a transcriptional repressor that plays a critical role in hematopoiesis, and its expression is negatively related to the prognosis of acute myeloid leukemia (AML) patients. We earlier reported a change in the metabolic state of hematopoietic stem cells upon *Gfi1b* deletion. Here we explored the role of Gfi1b in metabolism reprogramming during hematopoiesis and leukemogenesis. We demonstrated that *Gfi1b* deletion remarkably activated mitochondrial respiration and altered energy metabolism dependence toward oxidative phosphorylation (OXPHOS). Mitochondrial substrate dependency was shifted from glucose to fatty acids upon *Gfi1b* deletion via upregulating fatty acid oxidation (FAO). On a molecular level, Gfi1b epigenetically regulated multiple FAO-related genes. Moreover, we observed that metabolic phenotypes evolved as cells progressed from preleukemia to leukemia, and the correlation between Gfi1b expression level and metabolic phenotype was affected by genetic variations in AML cells. FAO or OXPHOS inhibition significantly impeded leukemia progression of *Gfi1b*-KO *MLL/AF9* cells. Finally, we showed that Gfi1b-deficient AML cells were more sensitive to metformin as well as drugs implicated in OXPHOS and FAO inhibition, opening new potential therapeutic strategies.

## Introduction

Hematopoiesis is a stepwise process tightly controlled by key transcription factors to produce and replenish the blood system. Although considerable progress in understanding hematopoiesis regulation has been achieved in past decades, open questions remain. Recent studies highlighted the role of metabolic regulation in fate transitions of hematopoietic stem cells (HSCs) and hematopoietic cells [1–4]. HSCs rely on glycolysis to maintain a quiescent state and protect from oxidative stress, while in response to stimuli, HSCs switch metabolism toward oxidative phosphorylation (OXPHOS), enter the cell cycle, and differentiate into downstream progenitors [1, 2]. Subsequent differentiating hematopoietic cells depend mainly on mitochondrial respiration to meet increased demand for energy and metabolic substrates [4–6]. The change in metabolites during the metabolic switch controls a series of epigenetic modifiers to determine the fate of hematopoietic progenitor cells (HPCs) by regulating key transcription factor activity [1, 2].

Acute myeloid leukemia (AML) is a group of hematological malignancies characterized by metabolic heterogeneity. Despite a high complete remission rate in AML patients, the 5-year overall survival is still poor, especially in patients over 60 years of age [7]. Accumulating data have highlighted the significance of metabolism reprogramming in the initiation, development, and drug resistance of leukemic cells [4, 8, 9]. Deregulation of cellular metabolism induced by transcription factors such as MYC supports the development of leukemia [10].

Growth factor independence 1B (GFI1B) plays a critical role in hematopoiesis by regulating the dormancy and proliferation of HSCs [11] and the development of erythroid and megakaryocytic cells [12–14]. Besides the regulation of normal hematopoiesis, low-level or loss of Gfi1b promotes AML development and negatively influences the prognosis of myelodysplastic syndrome (MDS)/AML patients [15]. However, the precise mechanism of how GFI1B regulates hematopoiesis and promotes AML development remains less explored. We previously showed that loss of Gfi1b leads to increased metabolic activation in HSCs [11]. Therefore, we investigated metabolic regulation of Gfi1b during hematopoiesis and leukemogenesis in human AML cell lines and mouse models, which could provide mechanistic insights into the hematopoiesis regulation and unveil potential therapeutic strategies for AML patients with low-level GFI1B.

## Material and methods

### Mouse models

*Gfi1b*^*fl/fl*^*MxCre*^*tg*^ and *Gfi1b*^*fl/fl*^*MxCre*^*wt*^ mice and polyinosinic-polycytidylic acid (poly(I:C), Invivogen Europe, Toulouse, France) treatment were performed as described [11, 15]. Concerning ex vivo study, lineage negative (Lin-) cells were depleted from total bone marrow (BM) cells using mouse lineage cell depletion kit (Miltenyi Biotec, Bergisch Gladbach, Germany), then treated with 1000 Unit/ml interferon-β (IFN β, Bio-Rad, Hercules, CA, USA) for 3 days. Lin-/c-kit+/Sca-1+ (LSK) cells were sorted with Aria III FACS Cytometer (BD Biosciences, San Jose, CA, USA) (Supplementary Fig. S1).

Induction of *MLL/AF9* leukemia and serial transplantations in mice were performed as described [15–17]. Mice were housed in specific pathogen-free conditions in the animal facility of University Hospital Muenster. All mouse experiments were performed with the approval of the local ethics committee (authorization numbers: 84–02.04.2015.A058 and 81–02.04.2020.A398).

### Metabolic measurements

Seahorse Extracellular Flux analysis was performed according to the manufacturer’s protocol as described [18, 19]. Briefly, cells in appropriate numbers were seeded in Poly-D-Lysine (Sigma-Aldrich, Darmstadt, Germany) coated XFe96 plates (Agilent Technologies, North Billerica, MA, USA) in the XF DMEM medium with optimized concentrations of supplements. The cell plate was incubated at 37 °C for 1 h in a non-CO_2_ incubator to reach ideal pH and temperature conditions. The measurements of oxygen consumption rate (OCR) and extracellular acidification rate (ECAR) were recorded at the basal levels and after sequential injections of inhibitors into Seahorse ports. The measurements were normalized with cell numbers using Hoechst staining after the seahorse measurements. Data analysis was performed with the Seahorse Wave software (Agilent Technologies, North Billerica, MA, USA). The information and optimized concentrations of inhibitors and cell numbers used are listed in Supplementary Tables S1 and S2.

### Statistical analyses

Statistical analysis was performed with GraphPad software (La Jolla, USA). Data are presented as mean ± standard deviation unless otherwise indicated. All experiments were repeated at least three times in more than triplicates unless otherwise indicated. Student’s *t*-test (two-sided, unpaired) was chosen for the comparisons unless otherwise indicated. *p* value <0.05 was considered statistically significant. *p* < 0.05 was marked with *, *p* ≤ 0.01 with **, and *p* ≤ 0.001 with ***.

### Online supplementary materials

Further information on the experimental procedures, tables, and figures are available in the Supplementary materials.

## Results

### *Gfi1b* deletion activates mitochondrial respiration in murine HPCs

**Fig. 1:**
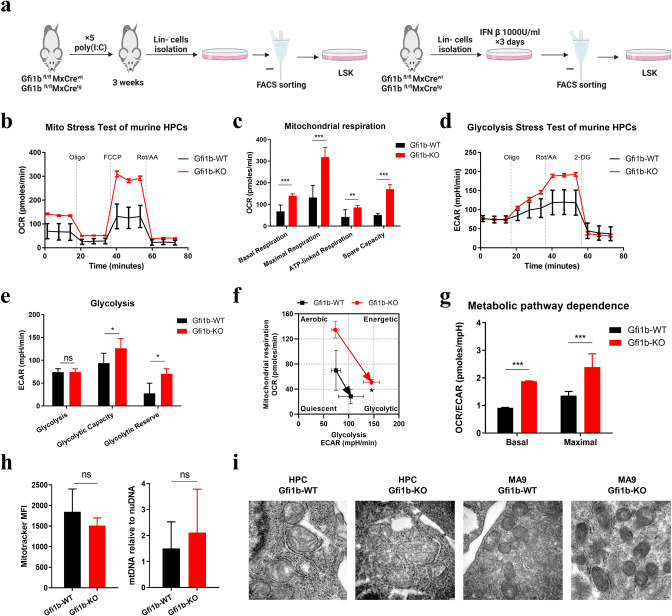
*Gfi1b* deletion activated mitochondrial respiration in murine hematopoietic progenitor cells (HPCs). **a** Left: schematic outline of *Gfi1b* deletion by poly(I:C) injection; mice were analyzed after about 3 weeks following the last injection. Right: schematic outline of IFN-β treatment ex vivo*;* LSK cells were sorted 7 days after IFN-β treatment. Seahorse Mito Stress test (**b**, **c**) and Seahorse Glycolysis Stress test (**d**, **e**) were performed with murine *Gfi1b-*WT/KO HPCs. **f** An XF PhenoGram was generated by plotting oxygen consumption rate (OCR) vs. extracellular acidification rate (ECAR) upon injection with oligomycin. **g** The dependence of energy metabolism of murine HPCs was calculated based on OCR/ECAR ratios under basal and maximal respiration conditions. **h** Mitochondria number was determined by MitoTracker Red (left) and mtDNA (right) in *Gfi1b*-WT/KO HPCs. **i** Representative electron microscopy images of mitochondria in murine *Gfi1b*-WT/KO HPCs or *MLL/AF9* leukemia cells (MA9) are shown. Scale bars, 100 nm.

To explore the role of Gfi1b in metabolic regulation in hematopoiesis, we used a conditional *Gfi1b* mouse model previously described [11, 15]. Cre-mediated *Gfi1b* excision was verified by genotype PCR and western blot (Supplementary Fig. S2a, b). About three weeks after poly(I:C) injection to induce *Gfi1b* deletion, we isolated Lin- cells and sorted LSK cells (Lin-/c-kit+/Sca-1+) for Extracellular Flux assay (Fig. 1a, left). Mito Stress test showed that *Gfi1b* knockout (*Gfi1b*-KO) cells had significantly higher basal and maximal OCR than *Gfi1b* wild type (*Gfi1b*-WT) cells, indicating that *Gfi1b* deletion activated mitochondrial respiration and increased respiration capacity in HPCs (Fig. 1b, c). ATP-linked respiration and spare respiration capacity were also markedly upregulated in *Gfi1b*-KO HPCs (Fig. 1c).

In addition, Glycolysis Stress test was performed to determine the effect of *Gfi1b* deletion on glycolysis. Basal glycolysis level did not change after *Gfi1b* deletion, but glycolytic capacity and glycolytic reserve increased in *Gfi1b*-KO cells (Fig. 1d, e). Cells regularly maintain metabolic plasticity by upregulating the glycolytic activity after OXPHOS inhibition to maintain stable ATP levels [20]. After oligomycin injection to inhibit mitochondrial respiration, we observed a significant increase in ECAR in *Gfi1b*-KO HPCs, suggesting increased metabolic plasticity in *Gfi1b*-KO HPCs (Fig. 1d, f).

Cells meet energy requirements through glycolysis and OXPHOS pathways. To further explore the effect of *Gfi1b* deletion on energy metabolism dependence, we examined OCR/ECAR ratios and observed significantly higher OCR/ECAR ratios under basal and maximal conditions in *Gfi1b*-KO HPCs, indicating that *Gfi1b* deletion shifted energy dependence toward OXPHOS (Fig. 1g). To further clarify whether metabolic change upon *Gfi1b* deletion in HPCs was mediated by the BM microenvironment, we isolated Lin- cells and deleted *Gfi1b* by adding IFN β in vitro (Fig. 1a, right). Similar effects on OXPHOS and glycolysis were observed; we thus concluded that Gfi1b-mediated changes in metabolism are cell-intrinsic (Supplementary Fig. S3).

To investigate how *Gfi1b* deletion affects mitochondrial respiration, we measured mitochondria numbers by flow cytometry with MitoTracker, mtDNA content by real time-PCR, and confocal fluorescent microscopy. Strikingly, all approaches showed no significant difference in mitochondria number between *Gfi1b*-KO and *Gfi1b*-WT HPCs (Fig. 1h and Supplementary Fig. S4), indicating increased OXPHOS in *Gfi1b-KO* HPCs potentially resulted from activated mitochondrial function, not from increased mitochondria number. Using transmission electron microscopy, we did not observe significant changes in mitochondrial morphology upon *Gfi1b* deletion (Fig. 1i). Together, our data demonstrated that *Gfi1b* deletion activated mitochondrial respiration and reprogramed the energy dependence in favor of OXPHOS in murine HPCs.

### *Gfi1b* deletion upregulates fatty acid oxidation (FAO) dependency

**Fig. 2:**
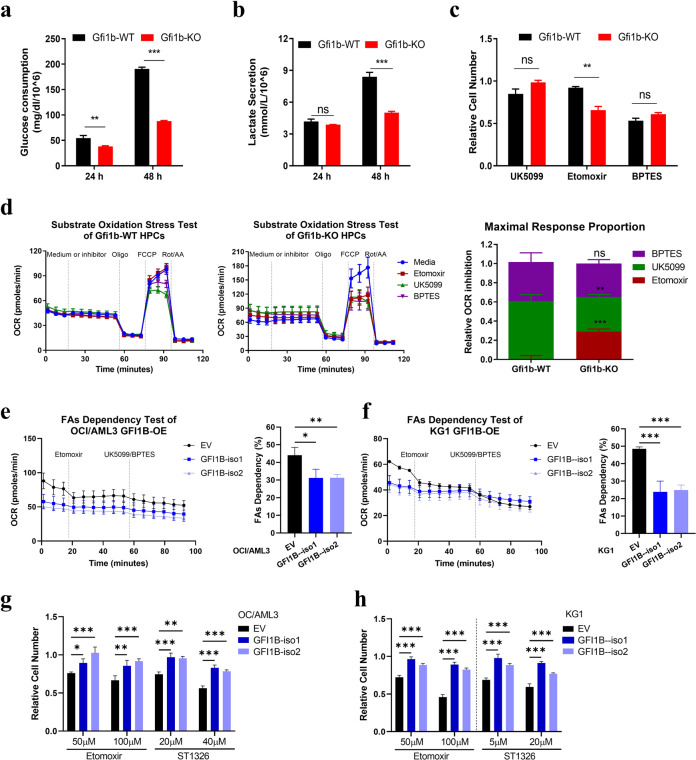
*Gfi1b* deletion increased fatty acid oxidation (FAO) dependency. **a**, **b** Glucose consumption and lactate secretion were measured every 24 h in murine HPCs ex vivo. **c** Murine HPCs were treated with 50 µM UK5099 to inhibit glucose oxidation, 80 µM etomoxir to inhibit FAO, or 40 µM BPTES to inhibit glutamine oxidation for 48 h. Cell numbers were determined and normalized to the solvent control. **d** The dependency on three primary mitochondrial substrates was determined by Seahorse Substrate Oxidation Stress test (4 µM etomoxir, 2 µM UK5099, or 3 µM BPTES). Maximal response proportions were calculated using the equation: target inhibitor response = media maximal OCR – target inhibitor maximal OCR; target response proportion = target inhibitor response / all inhibitors response. Data are representative of two independent experiments and are presented as mean ± SEM **e**, **f** Human AML cell lines OCI/AML3 (**e**) and KG1 (**f**) were transduced with lentiviruses to express two isoforms of GFI1B (GFI1B-iso1, -iso2) or empty vector (EV). Fatty acids (FAs) dependency was determined by first injecting 4 µM etomoxir to inhibit FAO, followed by the injection the two alternative pathways inhibitors (2 µM UK5099 and 3 µM BPTES). FAs dependency was calculated using the equation: FAs dependency (%) = (basal OCR – etomoxir OCR) / (basal OCR – all inhibitors OCR) × 100%. **g**, **h** OCI/AML3 (**g**) and KG1 (**h**) with GFI1B overexpression were treated with CPT1 inhibitors (etomoxir or ST1326) to inhibit FAO for 72 h, and cell numbers were determined and normalized to the solvent control.

To further investigate metabolism reprogramming induced by Gfi1b, we determined glucose consumption and lactate secretion rates, and found that *Gfi1b*-KO HPCs consumed less glucose and secreted less lactate than *Gfi1b*-WT HPCs (Fig. 2a, b). We thus speculated that *Gfi1b* deletion shifts substrate dependency from glucose to other fuels. To this end, we used specific substrate pathway inhibitors (UK5099, BPTES, or etomoxir) to inhibit the oxidation of three primary mitochondrial substrates (glucose, glutamine, or fatty acids (FAs), respectively), and determined the dependency on specific metabolic substrates. *Gfi1b*-KO HPCs were more sensitive to FAO inhibitor etomoxir rather than UK5099 or BPTES treatment with respect to cell proliferation (Fig. 2c). Apoptosis analysis revealed no difference in cell viability, suggesting that *Gfi1b*-KO cells potentially generate less ATP upon FAO inhibition and therefore divide less (Supplementary Fig. S5).

To assess the contributions of three primary substrates to mitochondrial respiration, we performed Substrate Oxidation Stress test. Upon *Gfi1b* deletion, increased sensitivity to etomoxir and resistance to UK5099 with respect to maximal mitochondrial respiration were observed in HPCs, indicating that loss of Gfi1b induced an elevated mitochondrial substrate dependency on FAs (Fig. 2d). To further validate the effect of GFI1B on FAs dependency, we transduced human AML cell lines OCI/AML3 and KG1 with two isoforms of GFI1B and performed Seahorse FAs Dependency test. GFI1B overexpression significantly reduced FAs dependency in OCI/AML3 and KG1 cells (Fig. 2e, f). Furthermore, in agreement with this data, GFI1B overexpression diminished the sensitivity to FAO inhibitors (etomoxir and ST1326) in OCI/AML3 and KG1 cells with respect to cell proliferation (Fig. 2g, h). These results suggested that GFI1B regulated mitochondrial substrate dependency on FAs.

### GFI1B regulates FAO-related genes expression

**Fig. 3:**
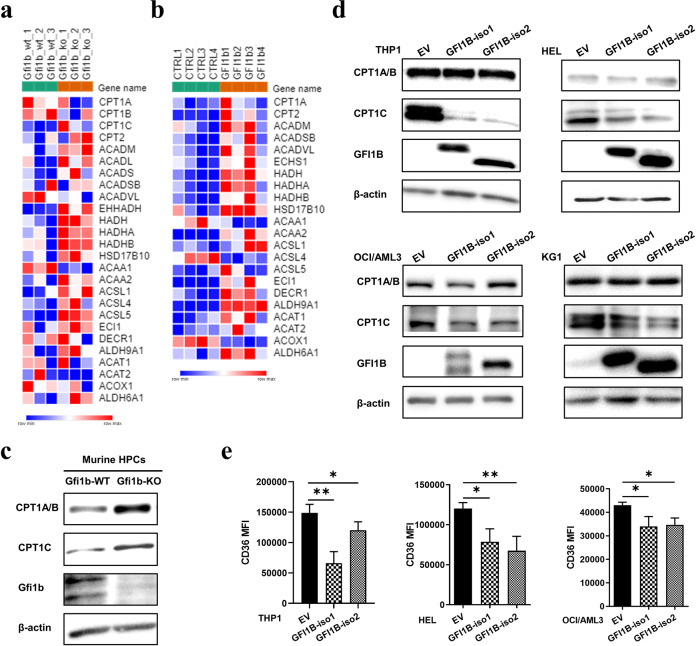
GFI1B regulated FAO-related genes expression. **a** Microarray analysis was performed with murine *Gfi1b-*WT/KO *NUP98/HOXD13* AML cells. Heatmap representing the genomic profiles of genes encoding FAO-related enzymes in *Gfi1b-*WT and *Gfi1b-*KO leukemia cells (*n* = 3). **b** Heatmap representing the proteins expression profiles of FAO-related genes in human control and dominant-negative mutant of GFI1B^Q287*^ platelets (*n* = 4) [21]. Box color is determined by low (blue) or high (red) expression levels. **c** The expression of different CPT1 isoforms in murine HPCs was determined by immunoblot; β-actin expression served as a loading control. **d**, **e** Human AML cell lines THP1, HEL, OCI/AML3, and KG1 were transduced with lentiviruses to express two isoforms of GFI1B (GFI1B-iso1,-iso2) or empty vector (EV). CPT1 expression was measured by immunoblot (**d**), and the expression of CD36 was determined by flow cytometry (**e**). The CD36 level in KG1 cells was undetectable.

FAO is the major catabolic pathway for lipids, fueling OXPHOS and the Krebs cycle. To explore how Gfi1b regulates FAs metabolism, we analyzed DNA microarray of blast cells from murine *NUP98/HOXD13* AML model [15]. Gene set enrichment analysis showed that loss of Gfi1b was associated with an enrichment of genes involved in FAO (Fig. 3a). Moreover, inherited mutation in GFI1B (GFI1B^Q287*^) disrupts its DNA binding zinc fingers, acting in a dominant-negative manner and mimicking the phenotype of GFI1B deficiency [21]. We thus performed proteomic analysis from published dataset to identify deregulated proteins in FAs metabolism in human GFI1B^Q287*^ platelets [21]. In concordance with microarray data, many proteins involved in FAO were enriched in GFI1B^Q287*^ platelets compared to healthy controls (Fig. 3b). Given that GFI1B mediates transcriptional repression by DNA binding, we next interrogated published ChIP-seq dataset from hematopoietic precursor cell line HPC-7 (GSE22178) for Gfi1b [22]. We found significant bindings of Gfi1b to several FAO-related genes (Supplementary Fig. S6). Hence, it is tempting to speculate that GFI1B epigenetically regulates the expression of different genes implicated in FAO.

To further substantiate our findings, we examined the expression of carnitine palmitoyltransferase I (CPT1), since this was the top candidate in our analysis and is associated with the translocation of FAs into mitochondria, a rate-limiting step of FAO [23]. Supporting our observation, the expression of CPT1 isoforms, CPT1A/B and CPT1C, was upregulated in *Gfi1b*-KO HPCs (Fig. 3c). Surprisingly, the expression of CPT1C, not CPT1A/B, was downregulated in various human AML cell lines with GFI1B overexpression (Fig. 3d). Furthermore, we determined the expression level of CD36 responsible for the uptake of FAs into the cytoplasm by flow cytometry. Supporting our hypothesis and in line with our observation, GFI1B overexpression significantly suppressed CD36 levels in various AML cell lines (Fig. 3e). Together, these data indicated an important role of Gfi1b in the regulation of FAO.

### GFI1B heterogeneously regulates mitochondrial respiration during AML development

To explore the metabolic regulation of Gfi1b in leukemic cells and metabolic evolution during leukemogenesis, we determined metabolic phenotypes of *Gfi1b*-KO cells in different leukemia stages in murine AML models.

**Fig. 4:**
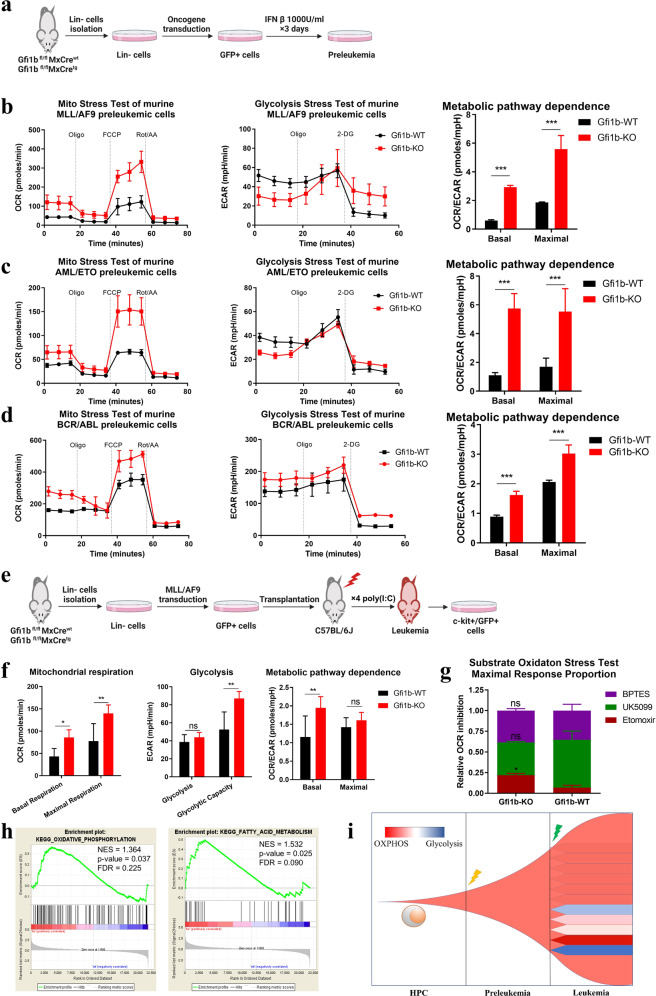
Gfi1b heterogeneously regulated oxidative phosphorylation (OXPHOS) and FAO in AML cells. **a** Preleukemias were generated by transducing oncofusion genes in murine lineage negative (Lin-) cells as indicated. Metabolic phenotypes and dependences of *MLL/AF9* (**b**), *AML/ETO* (**c**), or *BCR/ABL* (**d**) induced preleukemia were determined by Seahorse Mito Stress test and Glycolysis Stress test. **e** Schematic view of the murine *Gfi1b*-WT/KO *MLL/AF9* AML model. c-kit+/GFP+ blasts cells were isolated from the bone marrow of AML mice for the analysis. **f** Metabolic phenotypes and dependences of murine *Gfi1b*-WT/KO *MLL/AF9* AML cells were analyzed by Seahorse Mito Stress test and Glycolysis Stress test. A representative result from three independent experiments is shown (*n* = 4 mice). **g** Mitochondrial substrate dependency of murine *Gfi1b*-WT/KO *MLL/AF9* AML cells was determined by Seahorse Substrate Oxidation Stress tests (4 µM etomoxir, 2 µM UK5099, or 3 µM BPTES, *n* = 3 mice). Data are representative of two independent experiments and are presented as mean ± SEM. **h** Microarray analysis was performed with murine *Gfi1b-*WT/KO *NUP98/HOXD13* AML cells, and a gene set enrichment analysis (GSEA) of leukemic cells was performed. **i** Schematic view of hypothesis about metabolic evolution of *Gfi1b*-KO cells during AML development.

First, Lin- cells from *Gfi1b*^fl/fl^*MxCre*^*tg*^ or *Gfi1b*^fl/fl^*MxCre*^*wt*^ mice were transduced with different oncofusion genes (*MLL/AF9*, *AML/ETO*, or *BCR/ABL*) to generate preleukemic cells, and then treated with IFN β to induce *Gfi1b* deletion (Fig. 4a). Basal mitochondrial respiration and respiratory capacity in *Gfi1b*-KO preleukemic cells were significantly higher than those in *Gfi1b*-WT preleukemic cells expressing *MLL/AF9*, *AML/ETO*, or *BCR/ABL* (Fig. 4b–d, left). Of note, modest inconsistencies were observed in the glycolytic activity of *Gfi1b*-KO preleukemic cells expressing different oncofusion genes, potentially resulting from the deregulation of different pathways by oncofusion genes (Fig. 4b–d, middle). However, significantly elevated OCR/ECAR ratios were observed in *Gfi1b*-KO preleukemic cells with all oncofusion genes, suggesting energy production in *Gfi1b*-KO preleukemic cells mainly depended on OXPHOS (Fig. 4b–d, right). The data thus demonstrated that Gfi1b-deficient preleukemic cells shared metabolic phenotypes with Gfi1b-deficient HPCs.

Next, we determined metabolic phenotypes of leukemic cells isolated from a murine *MLL/AF9* AML model as described [15]. After AML development, c-kit+/GFP+ AML cells were isolated from BM as demonstrated (Fig. 4e). AML cells from *Gfi1b*-KO mice showed elevated mitochondrial respiration, respiration capacity, glycolytic capacity, and metabolic dependence on OXPHOS compared to *Gfi1b*-WT AML cells (Fig. 4f). Moreover, we noted remarkably divergent metabolic features within *Gfi1b*-KO and *Gfi1b*-WT AML groups (Supplementary Fig. S7), although all mice in each group were transplanted with the same donor-derived preleukemic cells. In addition, Substrate Oxidation Stress test showed that *Gfi1b*-KO AML cells were more sensitive to FAO inhibitor etomoxir than *Gfi1b*-WT AML cells (Fig. 4g), suggesting an increased level of FAO in *Gfi1b-*KO AML cells. To further validate our finding, whole-genome gene expression analysis was performed in the murine *NUP98/HOXD13* AML model as described [15], and loss of Gfi1b was associated with an enrichment of genes involved in OXPHOS and FAs metabolism (Fig. 4h). These data indicated that *Gfi1b* deletion upregulated OXPHOS and FAO in *MLL/AF9* AML mice.

Various factors, including genetic variation and microenvironment, collaboratively contribute to the metabolic phenotype of cancer cells [24, 25]. The divergent metabolic phenotypes in AML cells but not in preleukemic cells led us to explore whether the heterogeneity resulted from cell-intrinsic or -extrinsic factors during leukemogenesis. To exclude cell-extrinsic factors, we performed leukemia colony-forming units (L-CFU) assay (Supplementary Fig. S8). In line with in vivo data, *Gfi1b*-KO leukemic cells had a higher level of mitochondrial respiration than *Gfi1b*-WT, but notable metabolic heterogeneity was observed in the single colonies of Gfi1b-*KO* and Gfi1b-*WT* AML groups (Supplementary Fig. S8d). These data indicated that the metabolic heterogeneity resulted from cell-intrinsic factors.

Next, to explore whether the metabolic heterogeneity arises from the subsequent mutations during the leukemia development and further confirm our findings in vitro, we overexpressed GFI1B in various AML cell lines, and found that GFI1B overexpression significantly inhibited mitochondrial respiration and shifted metabolic dependence toward glycolysis in THP1, HEL, and OCI/AML3 cells (Supplementary Fig. S9a–c). In contrast, no significant effects on metabolic phenotype or dependence were observed in K562 and MOLM13 cells (Supplementary Fig. S9d, e). Collectively, these data indicated that metabolic properties evolved during leukemia progression, and genetic alterations influenced metabolism reprogramming induced by GFI1B in AML cells (Fig. 4i).

### Inhibition of FAO or OXPHOS impedes leukemia progression of *Gfi1b*-KO *MLL/AF9* cells

**Fig. 5:**
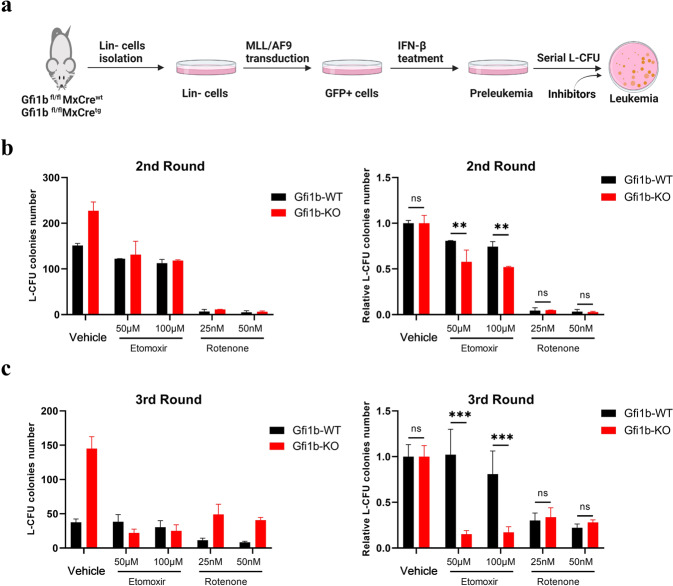
OXPHOS and FAO inhibition impeded leukemia progression of *Gfi1b*-KO *MLL/AF9* cells. **a** Overview of the experimental design for leukemia colony-forming units (L-CFU) assay in vitro. **b**, **c** Murine *Gfi1b*-WT/KO *MLL/AF9* preleukemic cells were plated in Methylcellulose medium subjected to OXPHOS inhibitor rotenone, FAO inhibitor etomoxir, or vehicle control, and colony counts were scored by ImageJ software at second plating (**b**) and third plating (**c**). Left, the absolute number of colonies; Right, the relative number of colonies to vehicle control.

We previously reported that loss/reduced expression of Gfi1b promotes AML development in different murine models of human AML [15]. The metabolites generated during metabolism reprogramming contribute to signaling functions, remodeling epigenome, and altering the expression of specific sets of genes, which can initiate and support cancer development [26]. Therefore, we performed serial L-CFU assay to investigate the function of metabolism reprogramming induced by *Gfi1b* deletion in leukemogenesis in the murine *MLL/AF9* AML model. After transducing Lin- cells with *MLL/AF9* to generate preleukemic cells, we plated them with OXPHOS inhibitor rotenone or FAO inhibitor etomoxir (Fig. 5a). We observed a substantial decrease in colony numbers of rotenone-treated *Gfi1b*-WT and *Gfi1b*-KO *MLL/AF9* cells, which supported our previous report about the high dependence on OXPHOS of *MLL/AF9* leukemia [27]. Untreated *Gfi1b*-KO leukemic cells generated more colonies than *Gfi1b*-WT cells, but *Gfi1b*-KO cells treated with etomoxir generated a similar number of colonies as *Gfi1b*-WT cells, indicating that FAO inhibition significantly impeded the rapid progression of Gfi1b-deficient leukemic cells (Fig. 5b, c). These results suggested that increased FAO and OXPHOS induced by *Gfi1b* deletion promotes leukemogenesis of *MLL/AF9* leukemia.

### Metformin inhibits the proliferation and c-Myc expression in Gfi1b-deficient cells

**Fig. 6:**
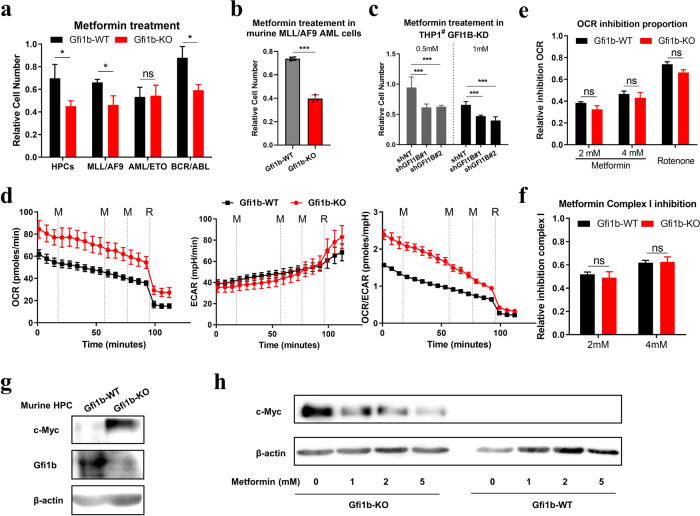
Metformin inhibited the proliferation and c-Myc expression in *Gfi1b*-KO cells. **a** Murine HPCs, and *MLL/AF9*, *AML/ETO*, or *BCR/ABL* preleukemic cells were treated with 2 mM metformin for 48 h, and cell numbers were determined. **b** Blast cells from *Gfi1b*-WT/KO *MLL/AF9* AML mice were treated with 1 mM metformin for 48 h, and the proliferation was determined ex vivo (*n* = 4 mice). **c** Human AML cell line THP1^#^ was transduced with two different shRNAs to target GFI1B (shGFI1B), non-target shRNA (shNT) as control. Proliferation was determined after metformin treatment for 48 h. **a**–**c** Cell numbers were normalized to the solvent control. **d** Mitochondrial respiration response to 2 mM metformin was determined by the Seahorse Mito Stress test in murine HPCs. OCR, ECAR, and OCR/ECAR ratio were measured upon sequential metformin (M) injections. Rotenone (R) was used to completely block complex I activity. **e** OCR inhibition proportion of metformin or rotenone was calculated. **f** Mitochondrial complex I activity inhibition by metformin was calculated as the percentage of the inhibition obtained with rotenone, set as 100%. **g** c-Myc expression was detected by immunoblot in murine *Gfi1b*-WT/KO HPCs. **h** Murine HPCs were treated with various concentrations of metformin for 48 h, and c-Myc expression was determined by immunoblot; β-actin expression served as a loading control.

Metformin was reported to impair the growth of cancer cells by inhibiting mitochondrial complex I [20, 28, 29]. Based on our observation, we next sought to develop a novel strategy for treating GFI1B-deficient AML patients. Except for *AML/ETO* preleukemic cells, murine *Gfi1b*-KO HPCs, *MLL/AF9*, and *BCR/ABL* preleukemic cells, and *MLL/AF9* AML cells were more sensitive to metformin treatment than *Gfi1b*-WT cells (Fig. 6a, b and Supplementary Fig. S10a, b). We further validated that metformin suppressed the proliferation of human AML cell line THP1^#^ with *GFI1B*-KD, without significant apoptosis induction (Fig. 6c and Supplementary Fig. S10c, d).

To explore the underlying mechanism, we measured mitochondrial respirations after metformin treatment for 24 h in HPCs. Basal and maximal OCR values were markedly inhibited in *Gfi1b*-WT and *Gfi1b*-KO cells (Supplementary Fig. S11a–d), but lower relative OCR inhibitions after metformin treatment in *Gfi1b*-KO cells were not observed compared to *Gfi1b*-WT cells (Supplementary Fig. S11c). To confirm our findings, we measured mitochondrial respiration of HPCs in response to metformin in real time. After sequential metformin (M) injections into the medium, we observed rapid and dose-dependent drops in OCR value and OCR/ECAR ratio and a rise in ECAR value in both *Gfi1b*-WT and *Gfi1b*-KO HPCs (Fig. 6d). No significant difference was found between *Gfi1b*-WT and *Gfi1b*-KO HPCs concerning OXPHOS inhibition and complex I inhibition (Fig. 6e, f). These data indicated that the high sensitivity of *Gfi1b*-KO cells to metformin was not caused by mitochondrial complex I inhibition.

Several studies have reported that metformin inhibits cancer cell proliferation by targeting c-Myc [30, 31]. By examining the published ChIP-seq datasets, we found that GFI1B binds to the promoter and enhancer regions of *c-Myc* [32, 33]. Therefore, we determined the relationship between Gfi1b and c-Myc by immunoblot. *Gfi1b* deletion increased the c-Myc level in murine HPCs, and GFI1B overexpression inhibited c-Myc expression in various AML cell lines (Fig. 6g and Supplementary Fig. S11e). To test the effect of metformin on c-Myc expression, we treated HPCs with various concentrations of metformin, and observed a dose-dependent downregulation of c-Myc protein level in *Gfi1b*-KO HPCs (Fig. 6h). These data suggested a potential mechanism for the high sensitivity of Gfi1b-deficient cells to metformin.

### AML cells with low-level Gfi1b are more sensitive to venetoclax and FAO inhibitors

**Fig. 7:**
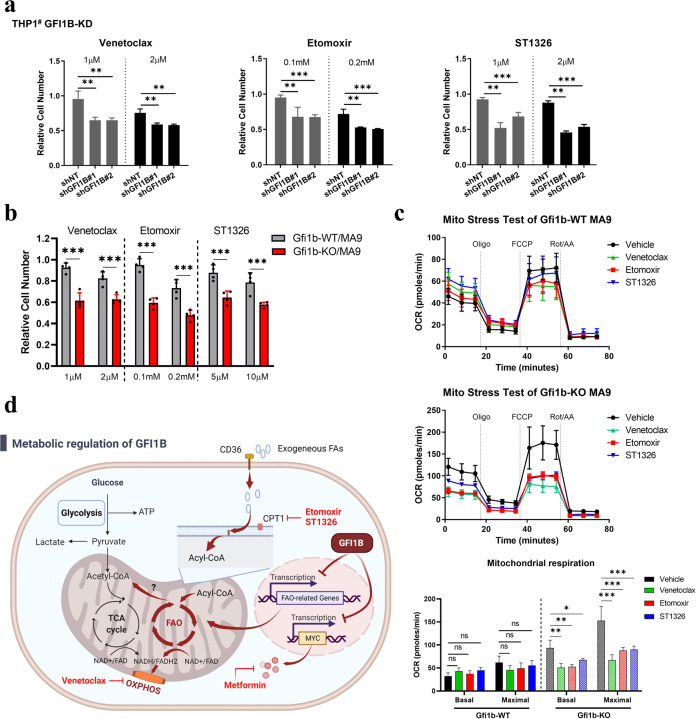
*MLL/AF9* AML cells with low-level Gfi1b were more sensitive to venetoclax and FAO inhibitors. **a** Proliferation of THP1^#^ cells with shRNAs to target GFI1B (shGFI1B) or non-target shRNA (shNT) were determined after venetoclax, etomoxir, or ST1326 treatment for 48 h. **b** Blasts cells from *Gfi1b*-WT/KO *MLL/AF9* AML mice were treated with venetoclax, etomoxir, or ST1326 for 48 h, and proliferation was determined (*n* = 4 mice). **a**, **b** Cell numbers were normalized to the solvent control. **c** Blasts cells from *Gfi1b*-WT/KO *MLL/AF9* AML mice were treated with 1 μM venetoclax, 0.1 mM etomoxir, or 5 μM ST1326 for 24 h, and mitochondrial respiration was determined (*n* = 3 mice). **d** Schematic depiction of metabolic regulation by GFI1B and potential targets for therapeutics in AML patients with low-level GFI1B.

Given that our observation of upregulated OXPHOS and FAO was made in Gfi1b-deficient AML cells, we tested the therapeutic effects of venetoclax and FAO inhibitors on the proliferation of AML cells with low-level Gfi1b. Venetoclax was recently reported to inhibit mitochondrial respiration independent of BCL-2 inhibition [34]; therefore, venetoclax and CPT1 inhibitors (etomoxir and ST1326) were employed. Treatment with venetoclax, etomoxir, or ST1326 led to marked suppression of proliferation in THP1^#^ with *GFI1B*-KD and murine *MLL/AF9* AML cells with *Gfi1b*-KO, without affecting cell viability (Fig. 7a, b and Supplementary Fig. S10d). Furthermore, we explored whether the high sensitivities arose from the inhibition of mitochondrial respiration. Murine *MLL/AF9* AML cells were treated with inhibitors for 24 h, and mitochondrial respiration was determined. Venetoclax, etomoxir, or ST1326 treatment induced significant decreases in basal and maximal OCR values in *Gfi1b*-KO AML cells rather than in *Gfi1b*-WT AML cells (Fig. 7c). These data suggested that venetoclax and FAO inhibitors could serve as potential therapeutics in AML patients with low-level GFI1B (Fig. 7d).

## Discussion

Gfi1b plays an essential role in hematopoiesis and mediates transcriptional repression by recruiting chromatin modifier complexes to the regulatory regions of the target [35], but the precise mechanism has yet to be elucidated. Here we show that in murine HPCs, *Gfi1b* deletion significantly upregulated mitochondrial respiration and FAO, and reprogrammed energy metabolism dependence toward OXPHOS and mitochondrial substrate dependency toward FAs. Via mutli-omics analysis, we found that Gfi1b regulated multiple FAO-related genes by direct DNA binding. However, future studies should investigate whether other mechanisms such as de-repression of c-Myc or rewired survival pathways induced by Gfi1b deficiency also contribute to metabolic changes. Gfi1b expression during hematopoiesis is strictly regulated by itself and other transcription factors [13, 36, 37]. Gfi1b is highly expressed in the HSCs, and its expression decreases in the progenitors and other differentiated cells except for megakaryocyte-erythrocyte progenitors and megakaryocyte precursors [38, 39]. Interestingly, metabolism reprogramming induced by Gfi1b is in line with metabolic phenotypes of hematopoietic cells with different Gfi1b levels during hematopoiesis. Future studies should examine metabolites changes regulated by Gfi1b and explore whether and how Gfi1b regulates hematopoiesis by metabolism reprogramming.

Emerging data highlight the role of OXPHOS and FAO in supporting the survival of chemoresistant AML cells and leukemic stem cells (LSCs) [8, 40]. Cytarabine-resistant AML cells exhibit increased FAO and OXPHOS gene signature [8, 41, 42]. Compared to HSCs, LSCs have a distinct metabolic profile and depend mainly on OXPHOS [40]. We previously showed that GFI1B deficiency is associated with inferior prognosis in MDS/AML patients and significantly increases LSCs number, partially resulting from epigenetic alterations of Gfi1b target genes [15], but additional mechanisms might contribute to this. Here, we found that *Gfi1b* deletion increased OXPHOS and FAO in AML cells, which is interestingly consistent with the metabolic features of shorter survival AML patients, cytarabine-resistant AML cells, and LSCs. Metabolism reprogramming induced by abnormal expression of transcription factors could enable cells to acquire metabolic profiles that supports the development, proliferation, and evasion of immune surveillance of cancers [24, 43]. We provided in vitro evidence that in *MLL/AF9* leukemia FAO or OXPHOS inhibition significantly impeded the rapid progression of *Gfi1b*-KO cells. Our findings suggest a potential mechanism for the inferior prognosis and high LSCs number in GFI1B-deficient AML patients. Future in-depth studies using epigenomics and metabolomics are needed to determine the molecular mechanism underlying the role of metabolism reprogramming by Gfi1b deficiency in leukemic transformation and progression.

Recent studies demonstrated that metabolic phenotypes of cancer cells are heterogeneous and context-specific, without fixed and broadly applicable liabilities [24, 25, 44]. Remarkable metabolic heterogeneity in AML mice transplanted with the same donor-derived preleukemic cells in our study reconfirmed the view in leukemias. Moreover, even though significantly elevated mitochondrial activity was observed in *Gfi1b*-KO HPCs and preleukemic cells, *Gfi1b*-KO AML cells showed divergent phenotypes and dependences. By determining metabolic phenotypes of single colonies in L-CFU assay and different AML cell lines with GFI1B overexpression, we confirmed that metabolic heterogeneity arose from genetic variations, not cell-extrinsic factors. Metabolic phenotypes and dependences evolve as cancers progress from preneoplasia to malignancies after acquiring subsequent mutations [24, 25]. Therefore, we hypothesize that in leukemic cells, the effect of some mutations on metabolic regulation is less potent than that of Gfi1b, and others are robust enough to mitigate the metabolic effect of Gfi1b loss; thus, the divergent effect of Gfi1b on metabolic phenotypes in AML cells reflect the effects of different mutations (Fig. 4i).

However, the role of GFI1B in leukemogenesis is still controversial. Some studies suggest GFI1B as a proto-oncogene possibly by supporting cell survival when abnormally expressed [45, 46], supported by the observation of apoptosis induction upon GFI1B inhibition in AML cell lines [35, 45, 47]. On the other hand, our group and others indicate a role of GFI1B as an oncosuppressor by driving differentiation toward the mature cells [15, 35, 48]. Based on our findings, we thus propose a hypothesis linking metabolism reprogramming by GFI1B with leukemogenesis: the role of GFI1B in leukemogenesis is developmental state-specific and mutation-dependent. In the early hematopoiesis stage, GFI1B acts as an oncosuppressor by driving differentiation toward specific hematopoietic lineages, and reduced GFI1B expression in concert with other signaling cascades blocks the differentiation and upregulates OXPHOS and FAO, which in turn promote LSCs augmentation and lead to an inferior prognosis and chemoresistance in AML patients. In the late hematopoiesis stage, an elevated level of GFI1B probably acts as a proto-oncogene by promoting cell proliferation and rewiring metabolism toward glycolysis, thus resulting in sensitivity to chemotherapy (Supplementary Fig. S12). Further research is required to provide information on this hypothesis.

As a safe drug widely used to treat diabetes, metformin is reported to inhibit cancer cell growth by inhibiting mitochondrial complex I [20, 28, 29, 49] or through other pathways [50]. We found an elevated sensitivity of Gfi1b-deficient cells to metformin treatment. By examining the response of mitochondrial respiration to metformin treatment, we confirmed that the high sensitivity to metformin was not caused by mitochondrial complex I inhibition in *Gfi1b*-KO cells. Combined with previous ChIP-seq data, we demonstrated that GFI1B represses c-Myc expression by directly binding the *c-Myc* promoter and enhancer. Furthermore, we observed a dose-dependent downregulation of c-Myc protein level after metformin treatment in *Gfi1b*-KO HPCs. These data suggest a possible mechanism for high sensitivity to metformin in low-level Gfi1b cells. Future studies should examine the therapeutic value of metformin in GFI1B-deficient AML in vivo and explore the underlying mechanism.

The BCL-2 inhibitor venetoclax was recently approved to treat newly diagnosed older AML patients due to high response rates [51]. Venetoclax can inhibit mitochondrial respiration independent of BCL-2 inhibition [34]. CPT1 inhibitors, including etomoxir and ST1326, could sensitize leukemic cells to the chemotherapeutic drugs [41, 52]. We found that venetoclax and FAO inhibitors exert better anti-leukemia effects by inhibiting mitochondrial respiration in GFI1B-deficient AML cells. Nevertheless, it should be noted that the metabolic phenotypes of the murine AML model and THP1^#^ used have been proved to be regulated by GFI1B in our study. Inconsistency of metabolic regulation by GFI1B across AML cells will limit target therapies for metabolic vulnerabilities. Therefore, technical advances in metabolic phenotyping and biomarker findings to predict therapeutic responses would benefit patients by allowing tailored therapies based on patient-specific cancer metabolism [24].

Collectively, we found that Gfi1b regulates OXPHOS, FAO, and c-Myc expression in hematopoiesis and leukemogenesis. However, metabolic regulation of GFI1B in AML cells is heterogeneous and genetic mutation-dependent. Furthermore, we showed potential therapeutic options to target GFI1B-deficient AML.

## Supplementary information

Supplementary Materials
