# Supplementary material for: GFI1B acts as a metabolic regulator in hematopoiesis and acute myeloid leukemia
Source: Leukemia. 2022 Jul 8;36(9):2196–207. doi: 10.1038/s41375-022-01635-9 (PMC9417998; doi:10.1038/s41375-022-01635-9)
Supplement: Supplementary file 1 — Supplementary Materials [file 41375_2022_1635_MOESM1_ESM.docx]

**Supplementary Materials**

**GFI1B acts as a metabolic regulator in hematopoiesis and acute myeloid leukemia**

**Supplementary Methods**

**Cell culture, retroviral and lentiviral transductions**

Human AML cell lines K562, THP1, HEL, OCI/AML3, KG1, and MOLM13 were obtained from DSMZ. THP1^#^ (kindly provided by Prof. Georg Lenz, University Hospital Muenster, Germany) was modified from THP1 to express a murine ecotropic receptor^1^. OCI/AML3 cells were cultured in complete alpha-MEM (Thermo Fisher Scientific, Bleiswijk, The Netherlands), and other cell lines were cultured in RPMI (Thermo Fisher Scientific, Bleiswijk, The Netherlands) with 10% fetal bovine serum (FCS, PAN-Biotech, Aidenbach, Germany). Murine hematopoietic progenitor cells (HPCs), preleukemic, and leukemic cells were maintained in IMDM (Thermo Fisher Scientific, Bleiswijk, The Netherlands) with 20% FCS, 10 ng/ml mIL-3, 10 ng/ml mIL-6, and 20 ng/ml mSCF (all from Miltenyi Biotec, Bergisch Gladbach, Germany). All media were supplemented with 100 U/ml penicillin and 100 mg/ml streptomycin (Thermo Fisher Scientific, Hennigsdorf, Germany), and all cells were maintained at 37°C with 5% CO_2_. Cell viability was evaluated using trypan blue (Sigma-Aldrich, MO, USA) staining.

shRNA-mediated *GFI1B* knock-down (KD) and retroviral transduction were performed as described^1,2^. Complementary DNA (cDNA)-mediated *GFI1B* knock-in and lentiviral transduction were performed as described^3–5^. Retrovirus production and transduction of *MLL/AF9*, *AML/ETO*, *BCR/ABL* constructs into murine lineage negative (Lin-) cells were performed as described^4^.

shRNA sequences and primer sequences used are listed in Supplemental Table S3. Plasmids information is listed in Supplemental Table S4.

**Flow cytometry analysis, sorting of LSK cells**

Flow cytometry was performed with Attune NxT Flow Cytometer (Thermo Fisher Scientific, Bleiswijk, The Netherlands), and data were analyzed using FlowJo (BD Biosciences, San Jose, CA, USA). For LSK cells sorting, Lin- cells were depleted from total bone marrow (BM), then applied to Aria III FACS Cytometer (BD Biosciences, San Jose, CA, USA). LSK cells gating strategy is shown in Fig. S1. All fluorochrome-conjugated antibodies and reagents used are listed in Supplementary Table S5.

**Glucose consumption and lactate secretion**

Glucose consumption and lactate secretion were measured from cell culture supernatant biochemically using an enzyme-based colorimetric kit (Biovision, San Francisco, CA, USA) according to the manufacturer's protocol, and the values were normalized to cell numbers at the time of supernatant collection.

**Immunoblot analysis**

Immunoblot was performed according to standard procedures as described^2,4^. Briefly, proteins were extracted from cells with PhosphoSafe Extraction Reagent (Merck KGaA, Darmstadt, Germany) and quantified using bicinchoninic acid assay (Thermo Scientific, Hennigsdorf, Germany). Proteins were electrophoresed on 8%-10% SDS-PAGE gel and transferred to a PVDF membrane (Merck Millipore, Darmstadt, Germany). Membranes were incubated with primary antibodies overnight at 4 °C and with secondary antibodies for 2 h at room temperature. Binding was detected using Radiance Plus Chemiluminescent substrate (Azure Biosystems, Dublin, CA, USA) and visualized using CHEMOSTAR ECL Imager (INTAS Science, Göttingen, Germany). All antibodies used are listed in Supplementary Table S6.

**Mitochondrial DNA (mtDNA) quantification**

mtDNA content was quantified by real-time PCR as described^6^. The primers used are listed in Supplementary Table S3.

**Confocal Microscopy Analysis**

Cells were harvested and washed with PBS (500 g, 4°C, 5 min). 50 nM MitoTracker Deep Red FM (Thermo Fisher Scientific, Hennigsdorf, Germany) was added for 30 min, followed by the addition of 5 ug/ml Hoechst33342 (62249, Thermo Fisher Scientific, Waltham, MA, USA) for10 min. Z-stacks of the samples were acquired using a Leica TCS SP8 confocal laser scanning microscope with an HC PL APO 63×/1.40 oil CS2 objective and Leica HyD hybrid detectors running on the LAS X version 3 software (Leica, Wetzlar, Germany). The excitation wavelengths of MitoTracker Deep Red and Hoechst33342 are at 644 nm and 405 nm, respectively. ImageJ software was used for the analysis of the images.

**Transmission Electron Microscopy (TEM)**

To visualize the ultrastructure of cell cultures, cells were centrifuged and fixed in 4% paraformaldehyde in 100 mM phosphate-buffered saline (PBS), pH 7.4 for at least 24 h. After extensive washing, all sections were treated with OsO4 and counterstained with uranyl acetate in 70% ethanol, dehydrated, and embedded in Durcupan resin (Fluka, Buchs, Switzerland). Resin blocks were made, and ultrathin sections were prepared with a Leica Ultracut S (Mannheim, Germany). Sections were adsorbed to glow-discharged Formvar carbon-coated copper grids. Images were taken using a Zeiss LEO 910 electron microscope (Zeiss, Oberkochen, Germany) equipped with a TRS sharpeye CCD Camera and manufacturer's software (Troendle, Moorenweis, Germany).

**Leukemia colony forming units (L-CFU) assay**

For the L-CFU assays, 5000 cells were plated in 0.4ml MethoCult media (M3434, StemCell, Vancouver, Canada), drugs were added at the desired concentration, and media was distributed in 24-Well plates with blunt-end needles. On day 10, L-CFU colonies were scanned and counted by ImageJ software**.**

**Supplementary Tables**

**Table S1. Chemicals used in Seahorse Extracellular Flux analysis and cell culture**

| **Chemicals** | **Catalog No.** | **Supplier** |
| --- | --- | --- |
| Oligomycin (Oligo) | 75351 | Sigma-Aldrich |
| FCCP | C2920 | Sigma-Aldrich |
| Rotenone (Rot) | R8875 | Sigma-Aldrich |
| Antimycin A (AA) | A8674 | Sigma-Aldrich |
| 2- Deoxy Glucose (2-DG) | D8375 | Sigma-Aldrich |
| (+)-Etomoxir sodium salt hydrate | E1905 | Sigma-Aldrich |
| UK5099 | PZ0160 | Sigma-Aldrich |
| BPTES | SML0601 | Sigma-Aldrich |
| ST1326 | 870853P | Sigma-Aldrich |
| Metformin Hydrochloride | PHR1084 | Sigma Aldrich |
| Venetoclax (ABT-199) | S8048 | Selleckchem |

**Table S2. Optimized cell numbers and inhibitor concentrations used in Seahorse XFe96 Extracellular Flux analysis.**

| **Cell type** | **Cells per well** | **Oligo** | **FCCP** | **Rot +AA** | **2-DG** |
| --- | --- | --- | --- | --- | --- |
| Murine HPCs | 150 000 | 2µM | 2µM | 500nM | 50mM |
| Murine preleukemic cells | 150 000 | 2µM | 2µM | 500nM | 50mM |
| Murine leukemic cells | 150 000 | 2µM | 2µM | 500nM | 50mM |
| THP1 | 100 000 | 1µM | 0.5µM | 500nM | 50mM |
| K562 | 100 000 | 1µM | 0.25µM | 500nM | 50mM |
| MOLM13 | 100 000 | 1µM | 0.25µM | 500nM | 50mM |
| HEL | 100 000 | 1µM | 0.25µM | 500nM | 50mM |
| KG1 | 100 000 | 1µM | 0.25µM | 500nM | 50mM |
| OCI/AML3 | 100 000 | 1µM | 0.25µM | 500nM | 50mM |

**Table S3. List of oligonucleotides and primers with name, sequences, and application.**

| **Name** | **Sequence (5'>3')** | **Application** |
| --- | --- | --- |
| shGFI1B_279 | GCCCTGTCCTTAGCACTCTAT | shRNA sequence #1 for *GFI1B-*KD |
| shGFI1B_355 | GGACCAGAACTTGGCCAGGAT | shRNA sequence #2 for *GFI1B-*KD |
| GFI1B_F | ATGCCACGCTCCTTCCTGG | Primers to amplify consensus coding sequence of human *GFI1B* |
| GFI1B_R | TCACTTGAGATTGTGCTGGCTC |  |
| Mus actB F | CGGCTTGCGGGTGTTAAAAG | Primers used in real-time PCR for murine mitochondrial DNA and nuclear DNA. |
| Mus actB R | CGTGATCGTAGCGTCTGGTT |  |
| Mus Cyt B F | CTTCATGTCGGACGAGGCTTA |  |
| Mus Cyt B R | TGTGGCTATGACTGCGAACA |  |

**Table S4. List of plasmids used in the study.**

| Name | Description | Reference |
| --- | --- | --- |
| pCL-ECO | Packaging construct for retrovirus production | Addgene plasmid # 12371. Ref.^7^ |
| pMIG-FLAG-MLL-AF9 | Retroviral construct to express MLL/AF9 oncofusion gene | Addgene plasmid # 71443. Ref.^8^ |
| MSCV-p210BCR-ABL | Retroviral construct to express BCR/ABL oncofusion gene | Addgene plasmid # 79248 |
| pMIG-AML-ETO | Retroviral construct to express AML/ETO oncofusion gene | Kindly provided by Dr. Michael Grez and Dr. Christian Wichmann^4,9^. |
| pRSMX-PG | Retroviral construct to express inducible shRNA | Kindly provided by Prof. Georg Lenz^1^. |
| pHIT-EAG | Envelope construct for retrovirus production of pRSMX-PG | Kindly provided by Prof. Georg Lenz^1^. |
| pHIT-60 | Packaging construct for retrovirus production of pRSMX-PG | Kindly provided by Prof. Georg Lenz^1^. |
| pCL6-IRES-EGwo | Empty lentiviral construct for cDNA expression | Ref.^4,5,10^ |
| pCD/NL-BH | Packaging construct for lentivirus production | Ref.^4,11^ |
| pcoPE | Human virus envelope expression plasmid | Ref.^4,11^ |

**Table S5. Antibodies and reagents used for flow cytometry**

| **Surface Receptor/Reagents** | **Catalog No.** | **Conjugates** | **Supplier** |
| --- | --- | --- | --- |
| CD117 (c-kit) | 135106 | PE | BioLegend |
| CD117 (c-kit) | 105811 | APC | BioLegend |
| CD117 (c-kit) | 105828 | Brilliant Violet 421 | BioLegend |
| CD4 | 100433 | PerCP | BioLegend |
| CD8a | 100711 | APC | BioLegend |
| Ly-6G/Ly-6C (Gr-1) | 108427 | PerCP | BioLegend |
| TER-119 | 116211 | APC | BioLegend |
| CD11b | 101211 | APC | BioLegend |
| CD45R/B220 | 103234 | PerCP | BioLegend |
| Lineage Cell Detection Cocktail-Biotin, mouse | 130-092-613 |  | Miltenyi Biotec |
| Streptavidin | 405229 | Brilliant Violet 605 | BioLegend |
| Streptavidin | 405214 | PerCP/Cyanine5.5 | BioLegend |
| Sca-1/ Ly6A/E | 105825 | APC/Cy7 | BioLegend |
| Sca-1/ Ly6A/E | 108105 | FITC | BioLegend |
| CD36 (anti-human) | 336207 | APC | BioLegend |
| Annexin V | 640920 | APC | BioLegend |
| Propidium Iodide | P-4170 |  | Sigma-Aldrich |
| MitoTracker™ Deep Red FM | M22426 |  | Thermo Fischer |

**Table S6: List of antibodies used for immunoblotting**

| **Antibody** | **Catalog No.** | **Supplier** |
| --- | --- | --- |
| β-Actin | 3700 | Cell Signaling |
| GFI1B | 5849 | Cell Signaling |
| c-Myc | ab32072 | Abcam |
| CPT1A/B | sc-393070 | Santa Cruz |
| CPT1C | 66072-1-Ig | Proteintech |

**Supplementary Figures**


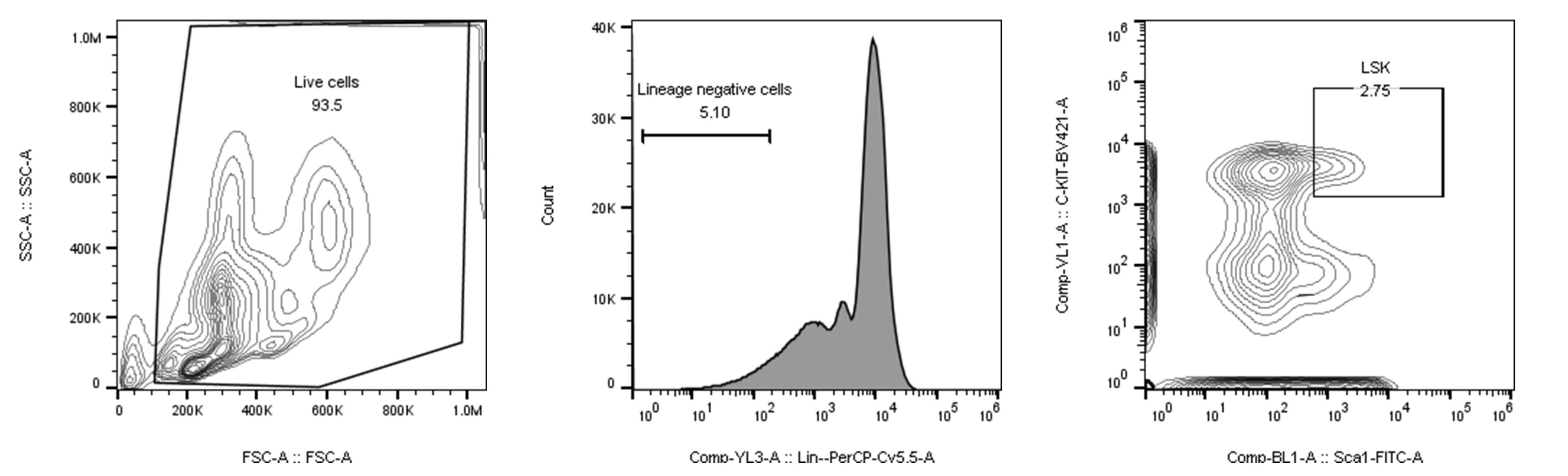


**Fig. S1: Representative gating strategy of LSK cells (HPCs) from murine total bone marrow (BM).**


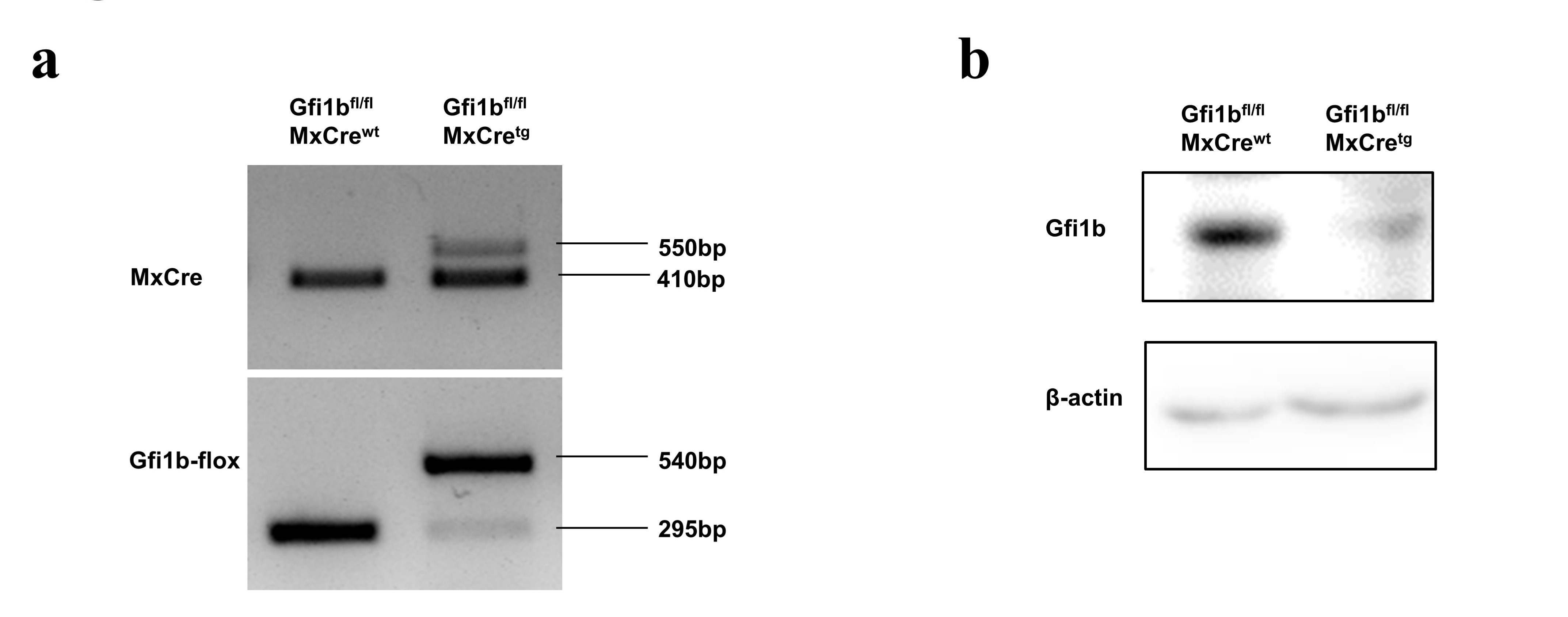


**Fig. S2: Cre-mediated *Gfi1b* excision in murine HPCs was verified by genotype PCR and immunoblot.**

Genomic DNA and protein were isolated from LSK cells of *Gfi1b*^fl/fl^*MxCre^tg^* and *Gfi1b*^fl/fl^*MxCre^wt^* mice after poly(I:C) injection or IFN-β treatment. Representative images of genotype PCR (**a**) and immunoblot (**b**) to confirm the deletion of *Gfi1b*. β-actin was used as a loading control.


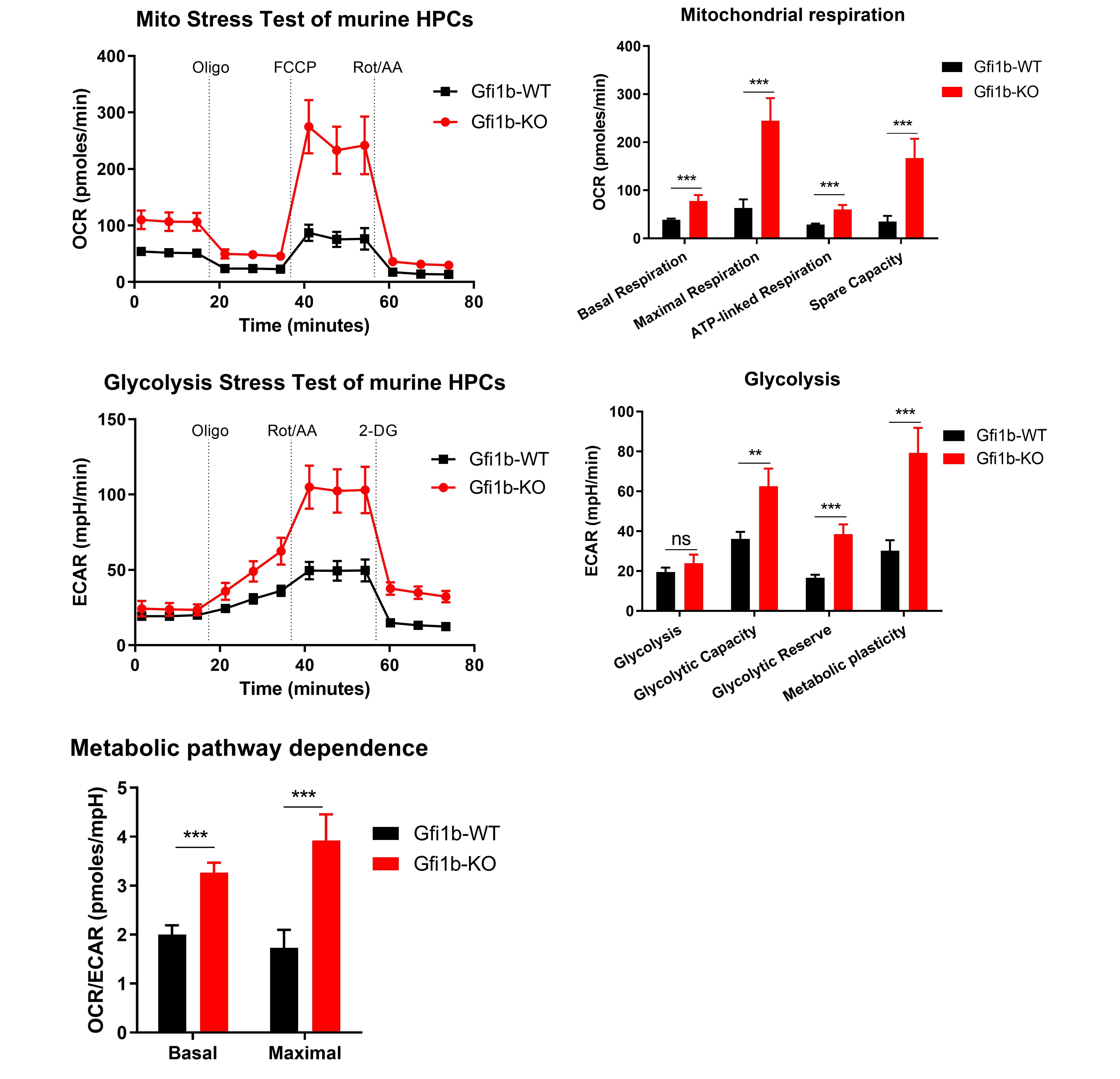


**Fig. S3: *Gfi1b* deletion activated mitochondrial respiration in murine HPCs *in vitro*.**

Lineage negative (Lin-) cells were isolated from the BM of *Gfi1b^fl/fl^MxCre^tg^* and *Gfi1b^fl/fl^MxCre^wt^* mice, and treated with 1000 U/ml IFN β for three days to delete *Gfi1b*. After 7 days of recovery period to wean off effects of IFN-β on HPCs, LSK cells were isolated by flow cytometry. Seahorse Mito Stress test and Glycolysis Stress test were performed to determine the metabolic phenotypes, and multiple parameters about mitochondrial respiration and glycolysis were calculated accordingly.


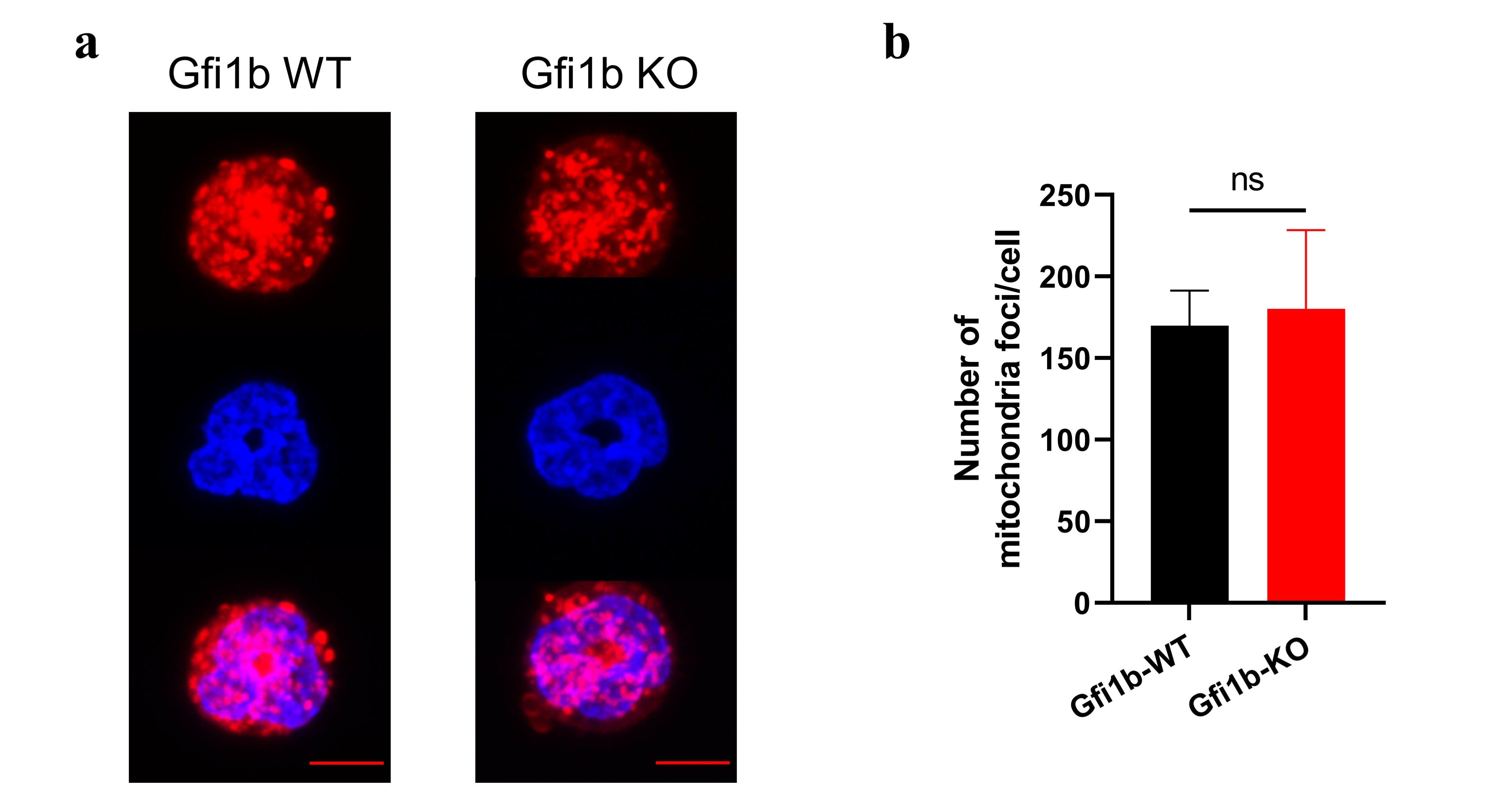


**Fig. S4: *Gfi1b* deletion did not increase mitochondrial number in murine HPCs.**

Murine HPCs isolated from *Gfi1b^fl/fl^MxCre^tg^* and *Gfi1b^fl/fl^MxCre^wt^* mice were stained with MitoTracker Deep Red for mitochondria (red) and Hoechst for nuclear (blue). **a** Representative images of mitochondria in HPCs. Scale bar, 5 µm. **b** Mitochondrial number per cell was counted with ImageJ (n=10-15).


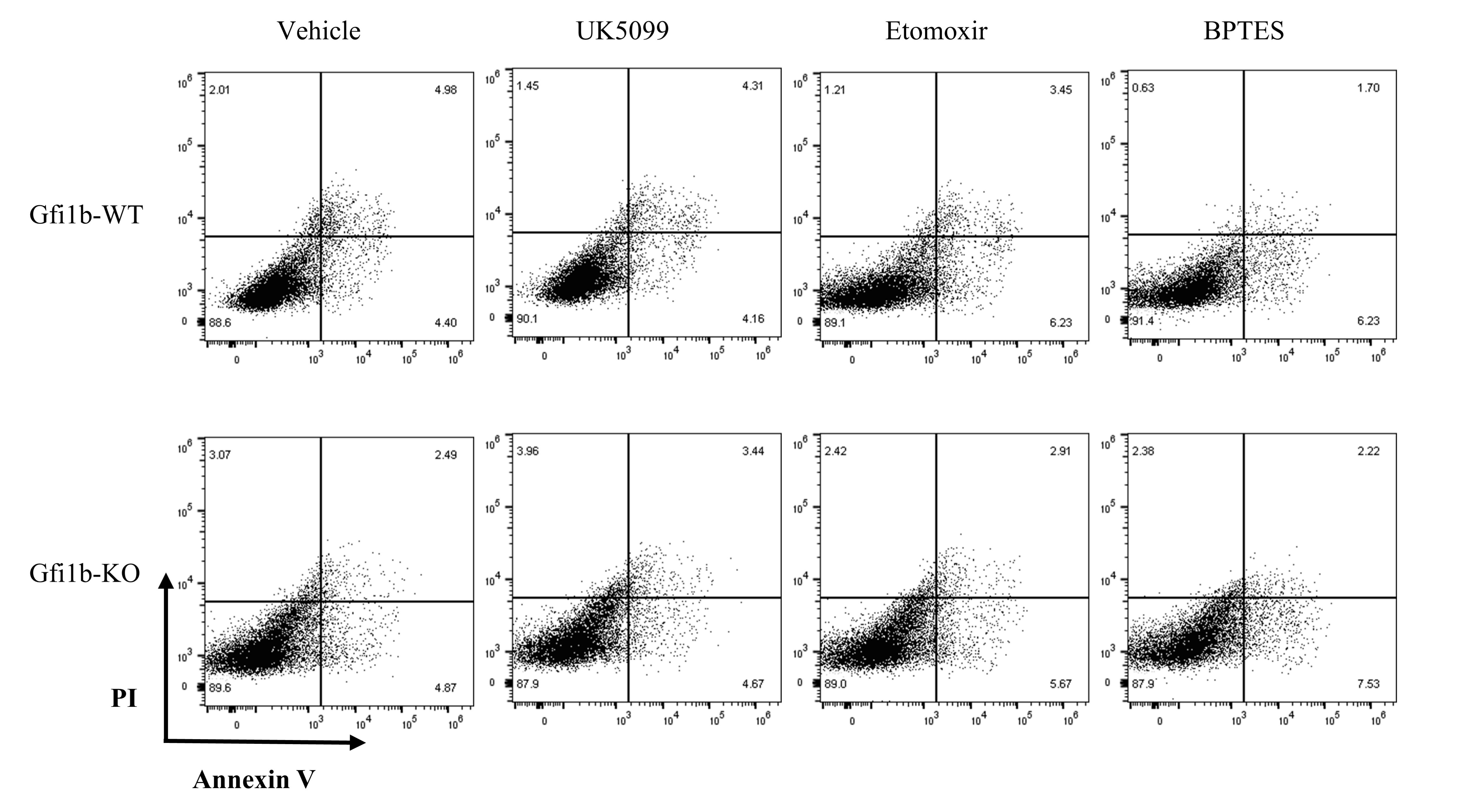
**Fig. S5: Treatments with substrate inhibitors did not induce apoptosis in murine *Gfi1b*-WT or *Gfi1b*-KO HPCs.**

Murine HPCs were treated with 50 µM UK5099 to inhibit glucose oxidation, 80 µM etomoxir to inhibit fatty acid oxidation (FAO), or 40 µM BPTES to inhibit glutamine oxidation for 48 h, and apoptosis was determined by flow cytometry. Representative images of flow cytometry from two independent experiments are shown.


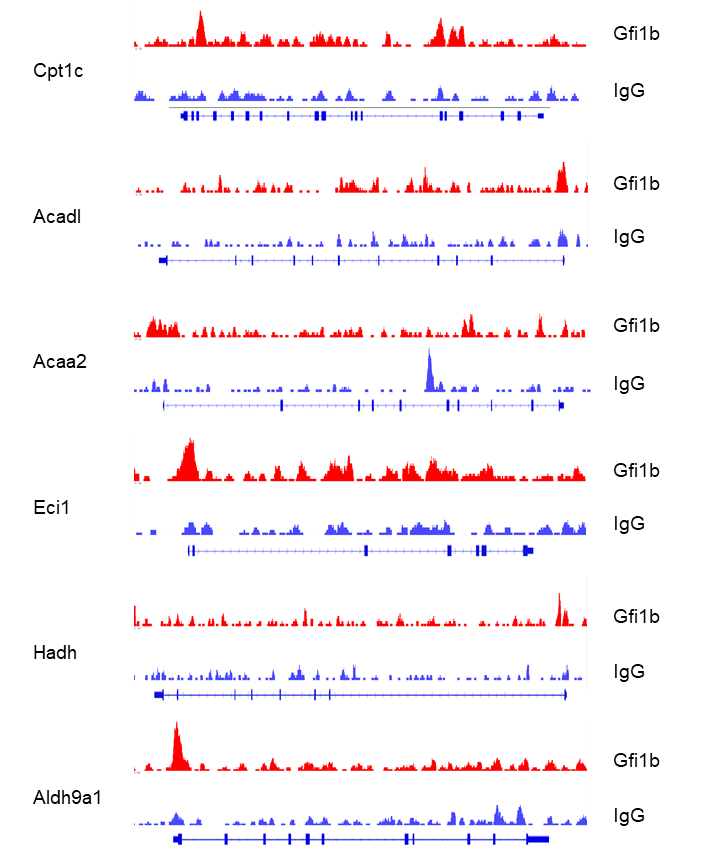


**Fig. S6: Gfi1b binds to FAO-related genes.**

Gfi1b occupancy (red) of FAO-related genes in murine HPC-7 cell line (GSE22178) was analyzed by ChIP-seq. IgG serves as control (blue).


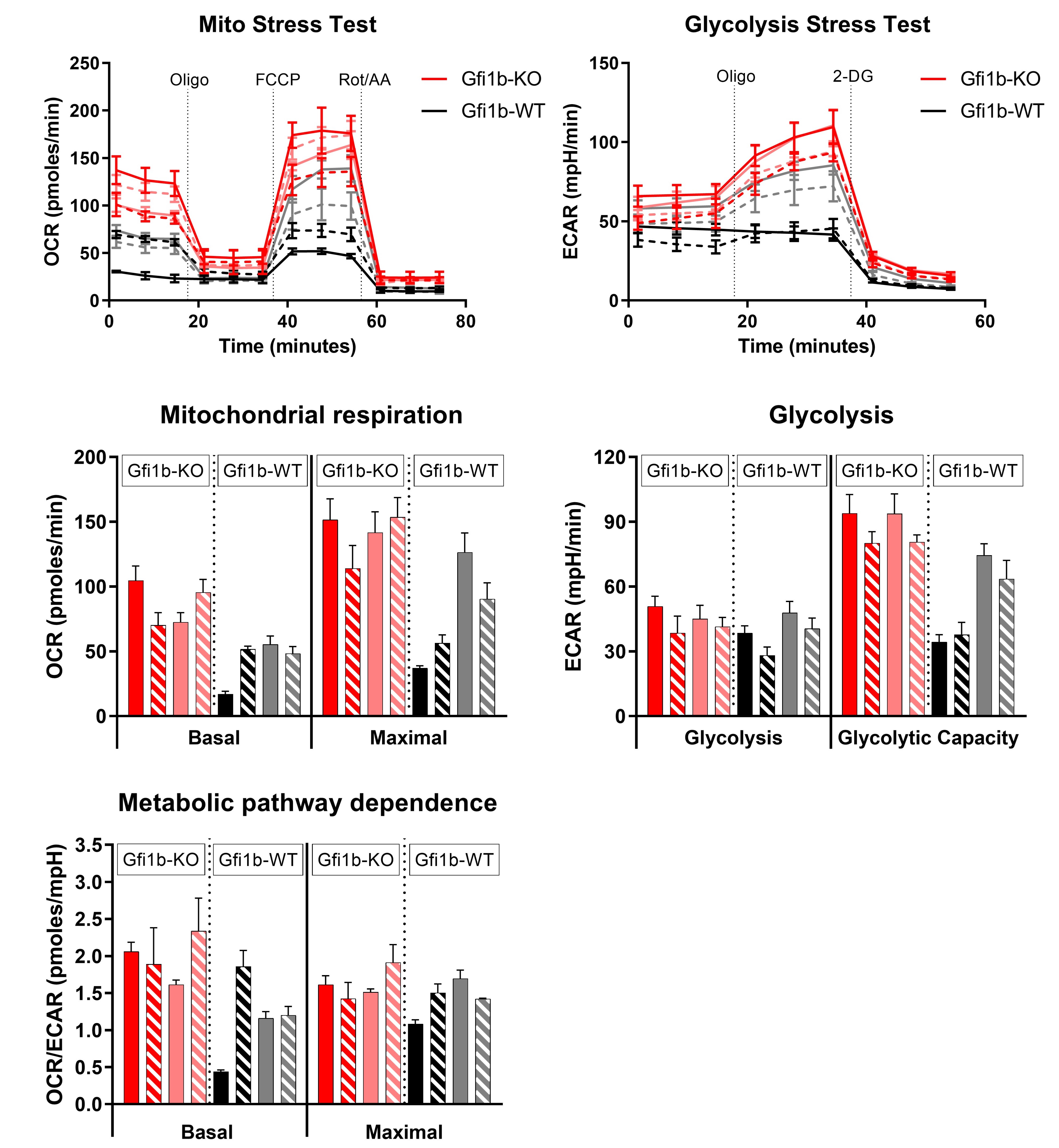


**Fig. S7: Metabolic heterogeneity was detected in *MLL/AF9* AML mice.**

Four C57BL/6J mice in each group were transplanted with *Gfi1b*-WT or *Gfi1b*-KO *MLL/AF9* preleukemic cells derived from the same mouse. After the AML development, c-kit+/GFP+ blast cells were isolated from BM, and metabolic phenotypes and dependences were determined by Extracellular Flux analysis. Each bar and line represent one individual mouse. Data are one representative result of three independent experiments.


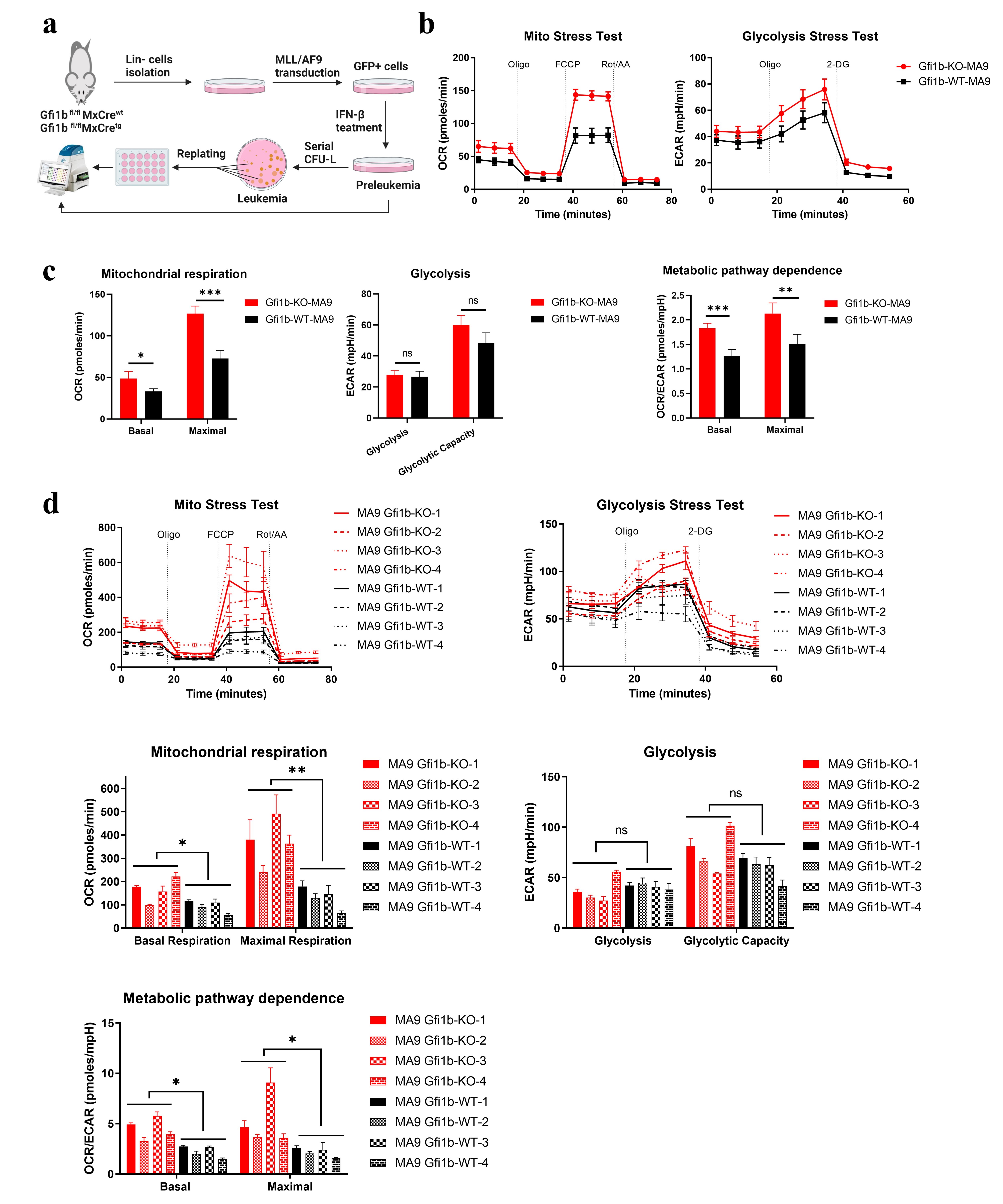


**Fig. S8: Metabolic phenotypes evolved during leukemogenesis *in vitro*.**

**a** Schematic outline of leukemia colony forming units (L-CFU) assays of murine *MLL/AF9* cells. **b**, **c** Metabolic phenotypes of preleukemia cells were determined before L-CFU assay. **d** After three round platings, single colonies were expanded, and the metabolic phenotypes were determined (n=4). Each bar and line represent one individual colony. Data are one representative result of two independent experiments.


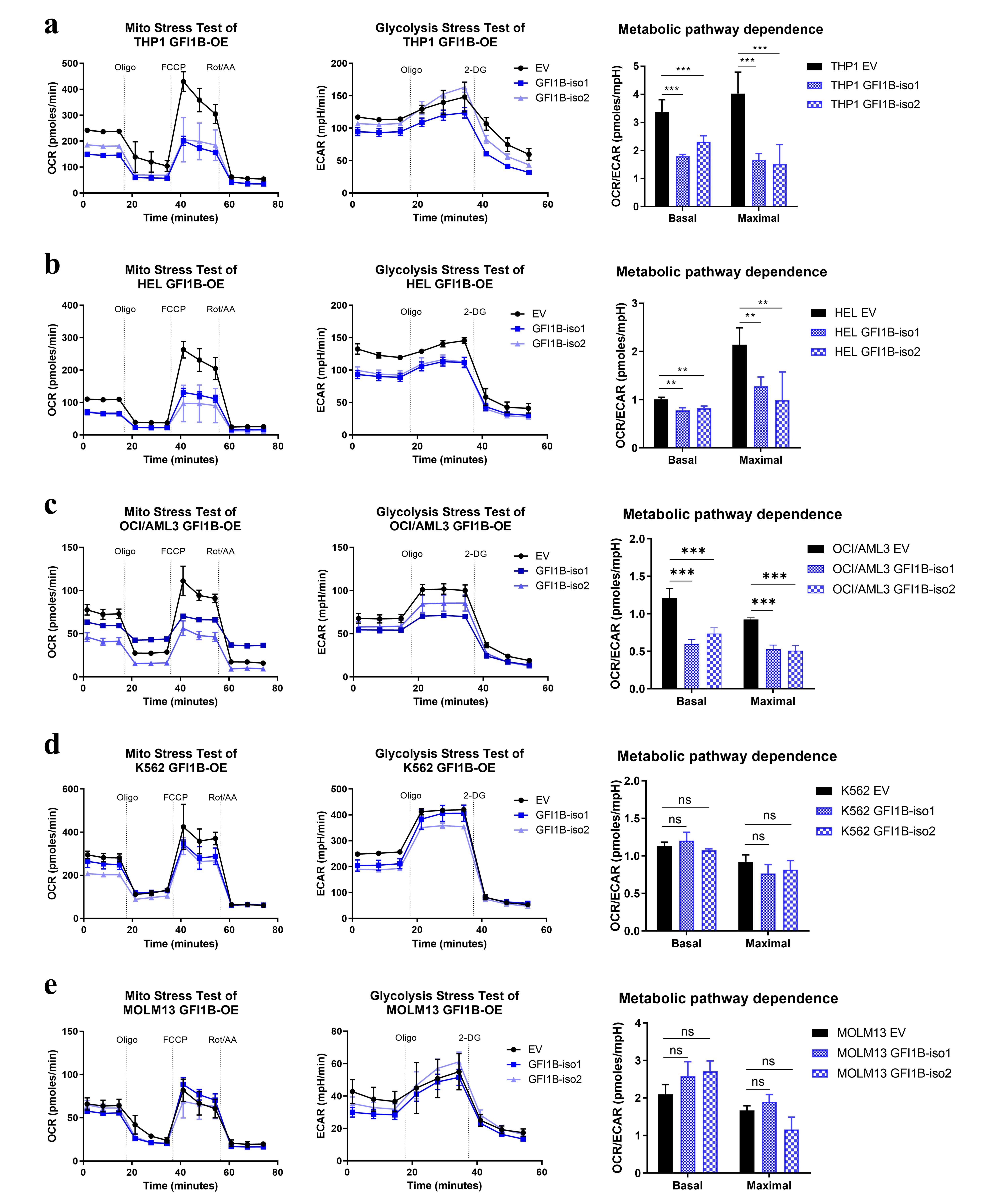


**Fig. S9: GFI1B heterogeneously regulated metabolic phenotype in human AML cell lines.**

Human AML cell lines THP1 (**a**), HEL (**b**), OCI/AML3 (**c**), K562 (**d**), and MOML13 (**e**) were overexpressed with two GFI1B isoforms (GFIB-iso1, -iso2) or empty vector (EV), and Seahorse Mito Stress test and Glycolysis Stress test were performed to determine the metabolic phenotypes and pathway dependences.


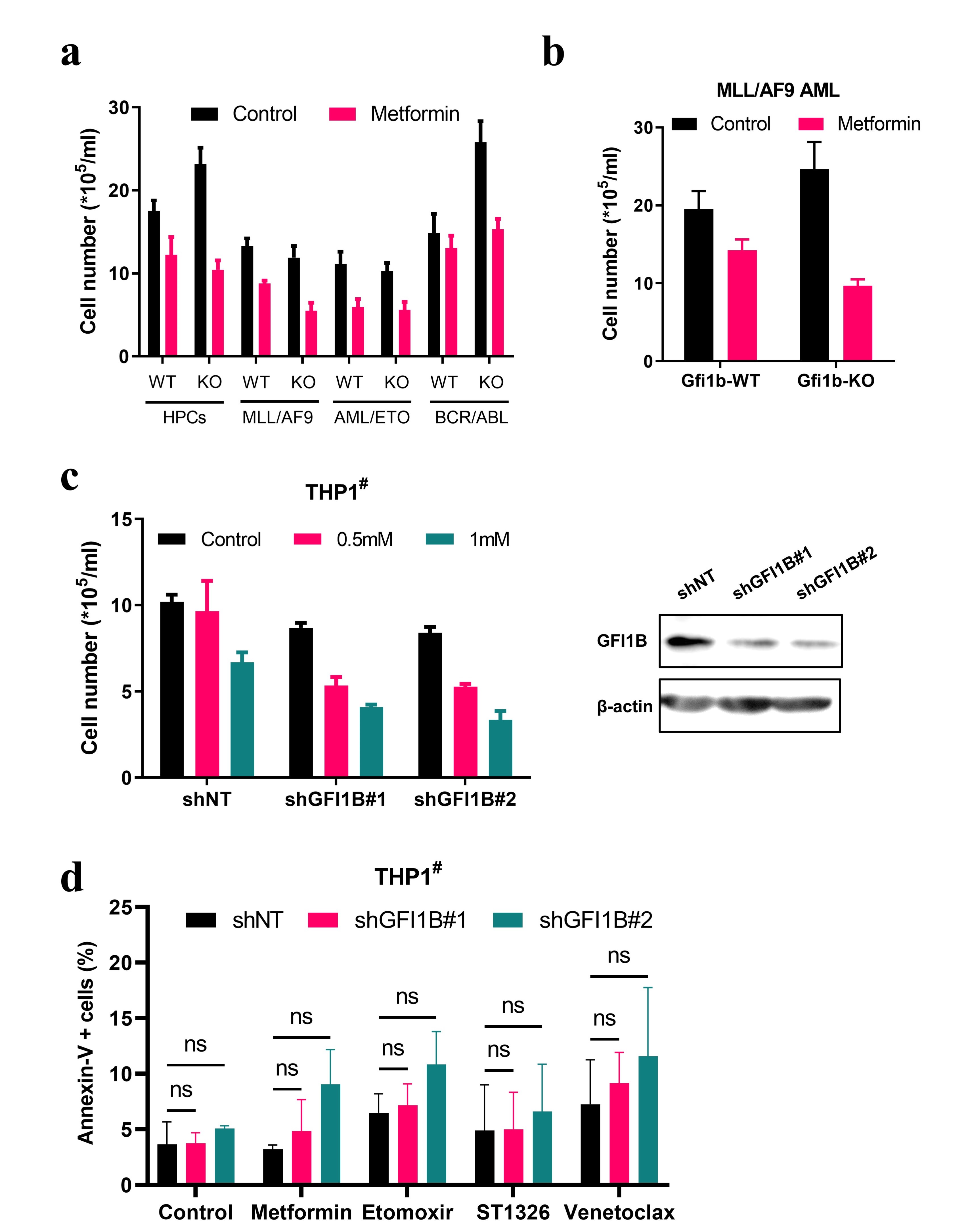


**Fig. S10: Metformin inhibited the proliferation without significant apoptosis induction in *Gfi1b*-KO cells.**

**a** Murine HPCs, and *MLL/AF9*, *AML/ETO*, or *BCR/ABL* preleukemic cells were treated with 2 mM metformin for 48 h, and cell numbers were counted. **b** Blast cells from *Gfi1b*-WT/KO *MLL/AF9* AML mice were treated with 1 mM metformin for 48 h, and the proliferation was determined *ex vivo*. (n=4 mice). **c** Human AML cell line THP1^#^ was transduced with two different shRNAs to target GFI1B (shGFI1B), non-target shRNA (shNT) as control. Proliferation was determined after metformin treatment for 48 h. **d** Apoptosis of THP1^#^ cells with shRNAs to target GFI1B (shGFI1B) or non-target shRNA (shNT) was determined after 2 mM metformin, 0.1 mM etomoxir, 1 µM ST1326, or 1 µM venetoclax treatments for 48 h by flow cytometry. The data are representative of three independent experiments.


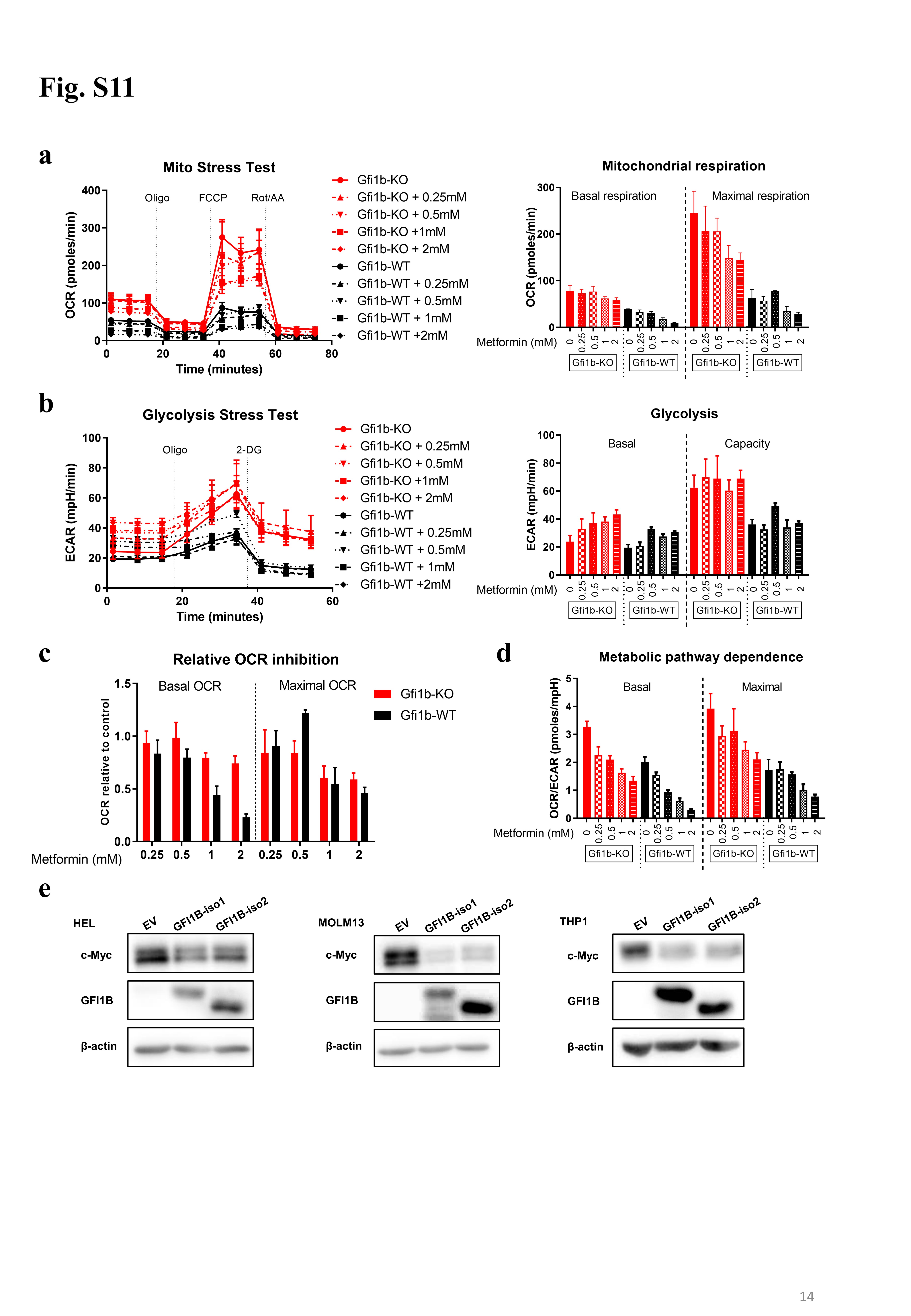


**Fig. S11: Metformin inhibited mitochondrial respiration and c-Myc expression in *Gfi1b*-KO cells.**

**a-d** HPCs isolated from *Gfi1b*-WT and *Gfi1b*-KO mice were treated with 2 mM metformin for 24 h *in vitro*, and mitochondrial respiration and glycolysis levels were determined by Extracellular Flux analysis. Mitochondrial respiration levels were measured by OCR values, and relative OCR inhibitions by metformin were calculated accordingly (**c**). **e** In human AML cell lines HEL, MOLM13, and THP1, two GFI1B isoforms (GFI1B-iso1, -iso2) or empty vector (EV) were overexpressed, and c-Myc expression was determined by immunoblot; β-actin expression served as a loading control.


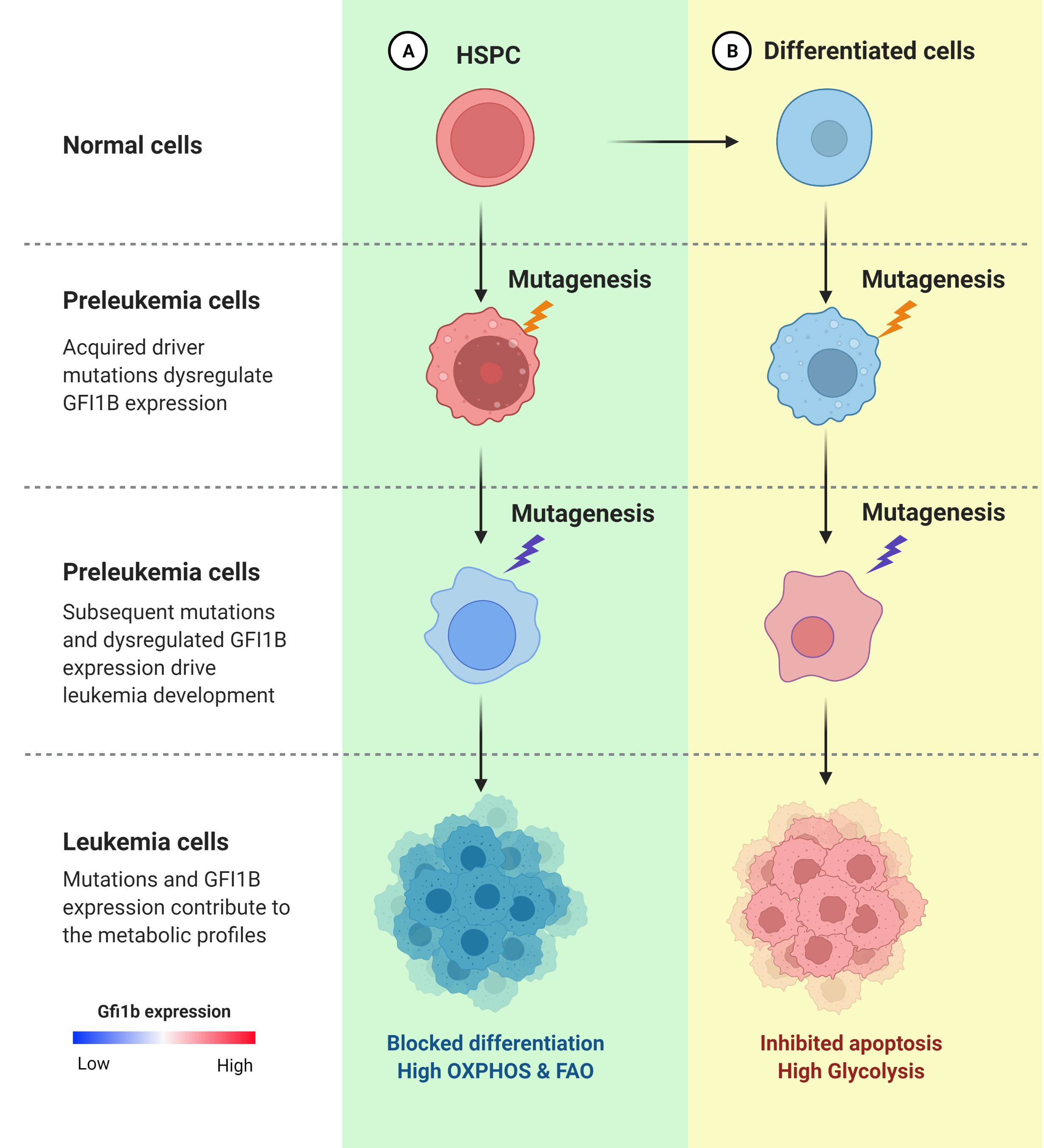


**Fig. S12: Proposed model: different roles of GFI1B in leukemogenesis.**

A: Early hematopoiesis stage; B: Late hematopoiesis stage. HSPC, Hematopoietic stem and progenitor cells. OXPHOS, Oxidative phosphorylation. FAO, Fatty acid oxidation.

**References**

1 Ngo VN, Davis RE, Lamy L, Yu X, Zhao H, Lenz G *et al.* A loss-of-function RNA interference screen for molecular targets in cancer. *Nature* 2006; **441**: 106–110.

2 Frontzek F, Staiger AM, Zapukhlyak M, Xu W, Bonzheim I, Borgmann V *et al.* Molecular and functional profiling identifies therapeutically targetable vulnerabilities in plasmablastic lymphoma. *Nat Commun* 2021; **12**: 5183.

3 Braun DA, Lovric S, Schapiro D, Schneider R, Marquez J, Asif M *et al.* Mutations in multiple components of the nuclear pore complex cause nephrotic syndrome. *J Clin Invest* 2018; **128**: 4313–4328.

4 Hönes JM, Thivakaran A, Botezatu L, Patnana P, Castro SV da C, Al-Matary YS *et al.* Enforced GFI1 expression impedes human and murine leukemic cell growth. *Sci Rep* 2017; **7**: 1–13.

5 Görgens A, Ludwig AK, Möllmann M, Krawczyk A, Dürig J, Hanenberg H *et al.* Multipotent Hematopoietic Progenitors Divide Asymmetrically to Create Progenitors of the Lymphomyeloid and Erythromyeloid Lineages. *Stem Cell Reports* 2014; **3**: 1058–1072.

6 de Almeida MJ, Luchsinger LL, Corrigan DJ, Williams LJ, Snoeck H-W. Dye-Independent Methods Reveal Elevated Mitochondrial Mass in Hematopoietic Stem Cells. *Cell Stem Cell* 2017; **21**: 725-729.e4.

7 Naviaux RK, Costanzi E, Haas M, Verma IM. The pCL vector system: rapid production of helper-free, high-titer, recombinant retroviruses. *J Virol* 1996; **70**: 5701–5705.

8 Saito Y, Chapple RH, Lin A, Kitano A, Nakada D. AMPK Protects Leukemia-Initiating Cells in Myeloid Leukemias from Metabolic Stress in the Bone Marrow. *Cell Stem Cell* 2015; **17**: 585–596.

9 Schanda J, Lee CW, Wohlan K, Müller-Kuller U, Kunkel H, Coco IQ Lo *et al.* Suppression of RUNX1/ETO oncogenic activity by a small molecule inhibitor of tetramerization. *Haematologica* 2017; **102**: e170–e174.

10 Wurm M, Schambach A, Lindemann D, Hanenberg H, Ständker L, Forssmann W-G *et al.* The influence of semen-derived enhancer of virus infection on the efficiency of retroviral gene transfer. *J Gene Med* 2010; **12**: 137–146.

11 Mochizuki H, Schwartz JP, Tanaka K, Brady RO, Reiser J. High-Titer Human Immunodeficiency Virus Type 1-Based Vector Systems for Gene Delivery into Nondividing Cells. *J Virol* 1998; **72**: 8873–8883.
